# Behavioral Signatures of Memory Resources for Language: Looking beyond the Lexicon/Grammar Divide

**DOI:** 10.1111/cogs.13206

**Published:** 2022-11-10

**Authors:** Dagmar Divjak, Petar Milin, Srdan Medimorec, Maciej Borowski

**Affiliations:** ^1^ Department of Modern Languages University of Birmingham Birmingham United Kingdom; ^2^ Department of English Language & Linguistics University of Birmingham Birmingham United Kingdom; ^3^ Department of Psychology, Centre for Applied Psychological Science Teesside University Middlesbrough United Kingdom

**Keywords:** Declarative, Procedural, Divided attention, Language, Memory, Dual task paradigm, Grammar, Lexicon

## Abstract

Although there is a broad consensus that both the procedural and declarative memory systems play a crucial role in language learning, use, and knowledge, the mapping between linguistic types and memory structures remains underspecified: by default, a dual‐route mapping of language systems to memory systems is assumed, with declarative memory handling idiosyncratic lexical knowledge and procedural memory handling rule‐governed knowledge of grammar.

We experimentally contrast the processing of morphology (case and aspect), syntax (subordination), and lexical semantics (collocations) in a healthy L1 population of Polish, a language rich in form distinctions. We study the processing of these four types under two conditions: a single task condition in which the grammaticality of stimuli was judged and a concurrent task condition in which grammaticality judgments were combined with a digit span task. Dividing attention impedes access to declarative memory while leaving procedural memory unaffected and hence constitutes a test that dissociates which types of linguistic information each long‐term memory construct subserves.

Our findings confirm the existence of a distinction between lexicon and grammar as a generative, dual‐route model would predict, but the distinction is graded, as usage‐based models assume: the hypothesized grammar–lexicon opposition appears as a continuum on which grammatical phenomena can be placed as being more or less “ruly” or “idiosyncratic.” However, usage‐based models, too, need adjusting as not all types of linguistic knowledge are proceduralized to the same extent. This move away from a simple dichotomy fundamentally changes how we think about memory for language, and hence how we design and interpret behavioral and neuroimaging studies that probe into the nature of language cognition.

## 1 Introduction

At first consideration, it might appear strange to think about something as prosaic as word forms as memories. Those who have *cycled the length of Hadrian's wall* will agree that these words conjure up all kinds of memories, ranging from getting soaked by a British summer shower to Roman history lessons at school. Yet, the word forms themselves, such as the past tense *cycled*, must be memories too, since memories harbor information that has been encoded, which is stored over time and which can be retrieved to influence future actions.

In this study of memory for language, we set out to determine the memory systems that underly specific dimensions of the knowledge that native speakers have about their mother tongue. Although there is a broad consensus that both the procedural and declarative memory systems play a crucial role in language learning and processing, the mapping between memory structures and linguistic types has not yet been explored systematically. The exclusive focus on syntax and the lexicon is at least in part due to the central position that syntax and the lexicon occupy in theories of language and language cognition: whereas generative, dual‐route models are heavily invested in a lexicon‐grammar split, for single‐route models such as usage‐based linguistics these are extremes of a continuum. In this study, we turn the tables: rather than selecting stimuli of types that fit theoretical assumptions about memory and language, we select stimuli of types that represent language to detect their memory signatures. These memory signatures help refine our understanding of the knowledge different memory systems subserve and enable us to arbitrate between generative and usage‐based models of language. To achieve this aim, we contrast knowledge of language, ranging from morphology and syntax to (lexical) semantics.

After a cursory introduction to memory, we move to the predictions that models of memory have made for language and discuss how these align with dominant linguistic theories. In Section 2, we go into detail about the experimental paradigm on which our linguistic study is based. We describe our results in Section 3, before discussing the implications of our findings for the study of memory structures for language and competing single versus dual‐route models of language in Section 4.

### 1.1 Co‐opted memory systems: Declarative and non‐declarative memory

Memory systems can be classified along a number of lines, the most common of which are the *information types* they process and the *operational principles* on which they rely (Squire, 2004; Squire & Wixted, 2011), alongside the *longevity* of the information they store (Baddeley, 1986; Baddeley, 2003; Squire, 2004). Declarative memory and non‐declarative or procedural memory are long‐term memory systems that can store an impressive amount of information for a long period of time (although, with time, some of it can become difficult to retrieve and appear “forgotten”).

Declarative memory is flexible, relational memory that can be accessed consciously and intentionally to guide behavior in new situations (P. J. Reber, Knowlton, & Squire, 1996). Learning by the declarative system is fast (even one‐trial learning is possible, see Squire, Knowlton, & Musen, 1993) but access to the information it harbors is slow and controlled (MacDonald, 2008; Richmond & Nelson, 2007), as well as fallible (Squire et al., 1993). Non‐declarative memory, although less well understood than declarative memory, is generally accepted to be acquired unconsciously. Access is also considered to be unconscious and non‐intentional, and the formation of procedural memories can be attested only through a change in behavior. Contrary to learning in the declarative mode, learning in the non‐declarative mode is slow, but access to the information is fast and automatic as well as reliable (MacDonald, 2008; Squire et al., 1993). Generally speaking, non‐declarative memory supports the more gradual learning of habits and skills alongside conditioning, habituation and sensitization, and priming (cf. Squire, 2004). The resulting memories encode rather inflexible, consistent relationships between stimulus and response, that is, between objects and/or events in the environment. The operational principle here is that of detecting and gradually extracting commonalities over time and across series of separate events.

The distinction between these two types of memory systems is supported by a firm body of evidence, showing that declarative and non‐declarative memory has distinctive neural correlates, that is, that they rest on different brain structures. Declarative memory relies on the medial temporal lobe, primarily on the hippocampal region (including the hippocampus, amygdala, and parahippocampal gyrus; cf. Squire, 1992; Squire & Zola‐Morgan, 1991; Squire, Bayley, & Smith, 2009), but other structures, such as the prefrontal cortex, the parietal cortex and the diencephalon also seem to play a role in storing and retrieving declarative memories (see, e.g., Chao, Haxby, & Martin, 1999; Rugg & Yonelinas, 2003; Wagner et al., 1998). Non‐declarative memory, of which procedural memory is one type, is less dependent on the medial temporal lobe and more on certain zones specialized for the acquisition of habits or conditioning and habituation/sensitization. For example, habits seem to rely on the striatum (cf. Mishkin, Malamut, & Bachevalier, 1984), while conditioning appears to be crucially dependent on the cerebellum (cf. Thompson & Krupa, 1994; Woodruff‐Pak & Lemieux, 2001).

Declarative memory is also known as explicit memory and non‐declarative memory as implicit memory or procedural memory (Squire, 1992), referring to the way information can be accessed or retrieved in these systems, that is, can it be declared and explicated or not. This signals a parallel, but not necessarily a perfect overlap between memory systems and modes of learning: declarative memory is the only memory system known to support explicit, knowledge‐based learning, described as “knowing that,” while non‐declarative memory underlies implicit motor, perceptual and cognitive skills, described as “knowing how” (P. J. Reber, 2008; Ryle, 1949 [2002]). However, there is evidence that declarative memory is also capable of supporting implicit learning (e.g., Moody, Bookheimer, Vanek, & Knowlton, 2004). The dissociability of implicit and explicit learning systems has long been established: there is general agreement that they involve different types of representations and are substantiated or represented in different parts of the brain (Cohen & Squire, 1980; Schacter, 1987; Squire & Kandel, 2009, but see Henke, 2010 for a more recent critique). And even though the systems are dissociable and have typically been studied in isolation, nearly all complex skills in the real world involve a mixture of explicit and implicit processes interacting in complex ways (A. S. Reber, 1989; Squire, 2004), leading to the development of integrated models of skill learning that take into account both implicit and explicit processes (Sun, Slusarz, & Terry, 2005).

Consequently, although there is evidence of the specialization of brain structures in supporting one or the other memory system, the existence of a firm distinction has been challenged, and brain areas that were previously thought to be exclusively involved in supporting one or the other memory system have been found to be less exclusive (Cabeza & Moscovitch 2013). At the functional level too, declarative and non‐declarative memory shows interdependence. Certain brain structures seem to be engaged in tasks that are otherwise expected to evoke one rather than another type of memory (e.g., the role of the prefrontal cortex in priming, habit formation and conditioning, and emotional conditioning in particular, cf. Dayan, 2007; Garcia, Vouimba, Baudry, & Thompson, 1999; Wagner, Koutstaal, Maril, Schacter, & Buckner, 2000; and in the formation of declarative memories, cf. Wagner et al., 1998; Brewer et al. 1998). This interdependence is, furthermore, subject to individual differences. In this respect, Poldrack et al. (2001) showed competition or trade‐off between the declarative and non‐declarative systems: participants differed in their relative dependence on the two systems and this relationship changed over the course of time, with declarative memory playing a more prominent role early in learning.

Summing up, there is a considerable amount of empirical evidence, both neuro‐anatomical and cognitive‐functional, which shows a significant degree of autonomy of the two types of long‐term memory. Given the complexity of these structures and the complexity of their respective “responsibilities,” however, a considerable overlap or interaction between the two is to be expected. The question that arises is which system handles language. In the absence of compelling evidence that the neurobiological bases of language are domain‐specific from birth, it is accepted that language depends on neurobiological substrates that once subserved or still subserve other areas of cognition, even if those systems may later (have) become specialized for language (Ullman, 2016, p. 953). In fact, evidence is accumulating that the cortical system that supports language is indeed highly specialized (for a comparison of the brain systems involved in language vs. music, arithmetic, and cognitive control, see Fedorenko, Behr, & Kanwisher, 2011).

### 1.2 Declarative and procedural memory: Predictions for language

The co‐optation of memory systems for language, and of declarative and non‐declarative memory in particular, has yielded a wide range of predictions for language. Much work on memory and language assumes a declarative/procedural divide^1^ (for a detailed account, see Ullman, 2004). In essence, it is stipulated that the declarative and procedural memory systems roughly underly the learning of lexicon versus grammar, respectively.

As mentioned above, the brain system underlying procedural memory handles rule‐based procedures (Knowlton & Squire, 1996), in particular those that involve detection of sequential and hierarchical structures. This property makes the procedural system ideal for supporting the learning and use of all subdomains of grammar that depend on sequences and hierarchies. In Ullman's model (Ullman, 2004, pp. 245–246), for example, procedural memory would handle syntax, (inflectional and derivational) morphology (for regulars and affixed irregulars), aspects of phonology (sound combinations), and possibly non‐lexical compositional semantics. Declarative memory handles idiosyncratic knowledge, which encompasses arbitrary bits of information and arbitrary associations. In language it has been argued to support lexical knowledge (Eichenbaum, 2004; Squire, 2004). In Ullman's model (Ullman, 2004, pp. 244–245), this lexical knowledge covers simple, non‐derivable words (because form‐meaning mappings are typically unmotivated), but also morphological irregularities. In addition, it hosts bound morphemes and knowledge of syntactic subcategorization frames. Declarative memory also harbors chunks (i.e., idioms and proverbs), which means that its content is not limited to individual items.

The proposal that each memory system subserves a different dimension of language, with the declarative system handling idiosyncratic knowledge, and the procedural system the sequencing of elements into more complex hierarchical structures, is by and large supported by behavioral and neurological evidence (for an overview, see Ullman, 2016). Neuroimagining studies of patients suffering from amnesia reveal lesions in brain structures subserving declarative memory (going back to patient H.M., see Squire & Wixted, 2011), while children with specific language impairment affecting syntax show atypical structure and function of brain areas subserving procedural memory (for a review, see Mayes & Morgan, 2015). Behavioral studies show correlations between either vocabulary learning abilities and learning abilities in declarative memory as captured by standard memory tests, or between grammar learning abilities and learning abilities in procedural memory, as tested by, for example, the serial reaction time (SRT) task (cf. Lum, Conti‐Ramsden, Morgan, & Ullman, 2014; Lum, Conti‐Ramsden, Page, & Ullman, 2012).

### 1.3 Predictions for language: Reconciling opposing linguistic theories

A lexicon‐grammar split reflects the assumptions of a generative account of language (Chomsky, 1965; Chomsky, 1995; Pinker, 1999), which dominated the linguistic scene during the second half of the 20th century, and continues to dominate work on the neuroscience of language (Dapretto & Bookheimer, 1999; M. Siegelman, Blank, Mineroff, & Fedorenko, 2019). Generative theory assumes pre‐existing but acquired abstract syntactic rules, devoid of meaning, which perform computational operations on memorized lexical items (for a discussion, see Divjak, 2019, p. 107). Simultaneously, generative theory sees, or used to see, the lexicon as rather uninteresting because it assumes that the lexicon contains everything that cannot be handled by rules and constraints: it is, in essence, a store of arbitrary labels. Famous is the jail‐metaphor used by di Sciullo and Williams (1987): the lexicon is a “jail” that contains the lawless items of language. Interesting are only the “ruly” parts of language, those whose combination is governed by the laws. Note here that “the lexicon” in generative accounts is not, or no longer, the same as the surface lexicon. Over the years, generative linguists have moved more and more into “the lexicon” and it now subsumes the primitives of the generative lexical system, plus bigger syntactically composed chunks subject to idiosyncratic interpretation and idiosyncratic morphological exponence. The grammar, conversely, incorporates generative mechanisms that involve significant amounts of non‐idiosyncratic regularity, often employing syntactic processes or processes that are analogous to syntactic processes (John Beavers, personal communication). Jackendoff (2002) blurred the boundaries between lexicon and grammar by advocating a store of memorized elements containing not only words plus phrasal units such as idioms and constructions, but also regular affixes and stems. This less rigid division of labor corresponds better with the *actual* division of labor proposed by the D/P model than a traditional generative view on language (Ullman, 2004, pp. 248–249).

The distinction between lexicon and grammar, be it rigid or lenient, that is pervasive in much work on memory and language does not mesh with views held by more recent usage‐based approaches to language. These approaches, inspired by single‐route models of language cognition (Rumelhart & McClelland, 1986), eschew a dual‐process view. On usage‐based accounts, the vast majority of our linguistic knowledge is underpinned by the implicit tallying of co‐occurrence that yields a distributional analysis of the language we are exposed to (Ellis, 2008, p. 125). Both grammar and lexicon are subject to this same process: usage‐based approaches naturally accommodate the finding that, at least initially, grammar and lexicon are one as children start from prefabricated chunks that combine words in specific forms (Tomasello, 1992; for differences with a generative view on language acquisition, see Ambridge & Lieven, 2011). Although this would suggest that the onus lies on declarative memory (Rumelhart & McClelland, 1986), over time, procedural memory is pressed into service, too: grammatical abstractions arise bottom‐up, that is, grammar is extrapolated from encounters with actual usage. Crucially, grammatical items and rules for their application can exist alongside prefabricated chunks that combine lexical items in specific forms. For example, even if users detect and store the English plural *‐s* which they need when combining the words *two* and *cup*, they may also store a partially or fully lexicalized chunk, for example, *two __s* or *two cups*. Usage‐based approaches have the assumption that grammar and lexicon are part of the same continuum built into their core: structures of either type (and of any size) convey meaning, be it more or less abstract.

The hypothesized grammar (rule) – lexicon (idiosyncrasy) opposition appears instead as a continuum on which linguistic abstractions can be placed as being more or less “ruly” or “defiant.” Furthermore, since linguistic knowledge is built bottom‐up, from exposure, linguistic information is variably entrenched in memory (for elaborate discussion, see Divjak, 2019). This process is generally linked to frequency of occurrence, with more frequent information expected to be more strongly entrenched (Langacker, 1987, p. 57). At the same time, high frequency of use would also lead to automatization (Bybee, 2006, p. 715), a claim that has not received much attention in the literature so far.

There is an abundance of psycholinguistic work on processing morphologically complex words that reflects this tension. In brief, inspired by the dichotomy between rules and exceptions (cf. Pinker, 1984, 1991), dual‐route models proposed two mechanisms for processing (e.g., pronouncing) regular words versus exceptions (see, e.g., the Dual Route Cascaded or DRC models in Coltheart, 1985; Coltheart & Rastle, 1994; Coltheart, Rastle, Perry, Langdon, & Ziegler, 2001). Connectionist single‐route models, developed within the parallel distributed processing (PDP) framework (e.g., Gonnerman, Seidenberg, & Andersen, 2007; Plaut & Gonnerman, 2000; Plaut, McClelland, Seidenberg, & Patterson, 1996; Seidenberg & Gonnerman, 2000), challenged this approach and instead proposed simultaneous or parallel processing such as phonological and semantic processing. Yet another take on this challenge is found in the *racing* model proposed by Baayen, Dijkstra, and Schreuder (1997). The model assumes two *parallel* rather than two *alternating* routes, implemented in a three‐layer spreading activation network. In a sense, it resembles Connectionist models, but conceptualizes the division of labor differently. The parallel dual‐route race model inspired fruitful debates on storage versus computation and the obligatoriness of decomposition in word processing.

Within psycholinguistics, both dual‐ and single‐route models evolved, with strong proponents on both sides (for dual‐route models, see, e.g., Hahn & Nakisa, 2000; Luzzatti, Mondini, & Semenza, 2001; Marcus, 1998; for single‐route models, among others, see Gonnerman et al., 2007; Harm & Seidenberg, 2004). Within linguistics, the distinction between grammar and lexicon continues to trigger debate, as witnessed by the marks it has left on the area of morphology. A morpheme can be defined in terms of its grammatical role (Marantz, 2013), or as a constructional schema (Booij, 2010), or the theoretical value of this construct can be denied altogether (Blevins, 2016).

## 2 This study

In this study, we investigate which memory systems subserve knowledge of different types of linguistic structures. Knowledge about the characteristic memory signatures for each of these different types of linguistic knowledge can also be used to arbitrate between dual‐ and single‐route linguistic theories.

Existing behavioral research in the area derives support for the declarative/procedural split between lexicon and grammar from a correlation between performance on tasks measuring procedural memory and syntactic learning ability, and declarative memory and lexical learning ability. In a departure from this practice, we measure the correlation between (either of) these memory systems and performance on a language task directly. The lure of a lexicon‐grammar split was no doubt strengthened by the focus of memory research on a formally simple language such as English that obscures the interdependence of grammar and lexicon. It has been suggested that an approach that separates lexicon from grammar might not extend well to morphologically complex languages (Kidd & Kirjavainen, 2011): with nouns being marked for case and verbs being marked for tense, mood, and aspect, grammar blends imperceptibly into the lexicon (and can no longer be distinguished at the neural level, see Fedorenko, Blank, Siegelman, & Mineroff, 2020). For this reason, we use data from a healthy population of L1 speakers of Polish, a morphologically rich Slavonic language, to pit the processing of morphological, syntactic, and lexical semantic information against each other.

We use a dual task paradigm, which is known to affect access to declarative and procedural memory differently, to test which dimensions of language knowledge are likely subserved by procedural or implicit memory and which ones depend more on declarative or explicit memory. The dual task paradigm contrasts a full‐attention condition, in which only a main task is executed, with a divided attention condition in which execution of the main task is paired with a concurrent task. If two tasks that tap into the same resources are performed simultaneously, performance will be impaired. If the tasks do not tap into the same resources, there should not be any effect on task performance. The dual‐task paradigm is known from studies investigating the role of (divided) attention on encoding and retrieval processes in human memory in general but also from studies investigating working memory (WM) more specifically.

The effects of divided attention on memory have been studied and probed extensively. Overall, it was found that divided attention at encoding is associated with large reductions in memory performance, but only small increases in response times (RTs); conversely, divided attention at retrieval yielded small or no reductions in memory but large increases in RT (Craik, Govoni, Naveh‐Benjamin, & Anderson, 1996). Going into more detail about the nature of the memory systems, Mulligan (1997) and Wolters and Prinsen (1997), among others, found that when WM is loaded by distractions or multitasking, explicit memory is affected, while implicit memory is left virtually unaffected (see Jimenez, 2003 for an overview and Spataro, Cestari, & Rossi‐Arnaud, 2011 for a meta‐analysis). Recent functional Magnetic Resonance Imaging work by Foerde, Knowlton, and Poldrack (2006) showed a fundamental difference in the sensitivity of the declarative and procedural memory systems to distraction and confirmed that declarative learning is disrupted by performing a secondary task at encoding while habit learning is not. The effects of divided attention at retrieval on memory systems have received less attention but recent findings in this area by Prull, Lawless, Marshall, and Sherman (2016) suggest that, in this case too, explicit memory would be affected while implicit memory would remain virtually unaffected by divided attention.

In other words, existing work has shown a differential impact of single‐ versus dual‐task conditions on canonical declarative and explicit versus procedural and implicit memory tasks; this difference is thought to be due to the fact that declarative memory and WM share resources. Many divided attention studies involving language have been run over the past three decades in order to study the central executive and its slave systems (including but not limited to Baddeley, Lewis, Eldridge, & Thomson, 1984; Craik et al., 1996; Fernandes & Moscovitch, 2002; Gordon, Hendrick, & Levine, 2002; Waters, Caplan, & Yampolsky, 2003). Although findings in the area of WM are equivocal (see Caplan & Waters, 1999 for discussion of the task and Caplan & Waters, 1990 as well as Varkanitsa & Caplan, 2018 for early and recent overviews of the findings) and opinions continue to diverge about the subsystems that need to be posited to explain the findings (see Conway, Kane, & Bunting, 2005; Doherty et al., 2019), there is general agreement that the brain regions that support the encoding and retrieval of declarative memories are also involved in processes handled by WM (Blumenfeld & Ranganath, 2007). Accessing declarative memory thus puts demands on WM, and hence, loading WM should affect access to the knowledge held in or processes governed by declarative memory (cf. Foerde et al., 2006).

The lack of executive control needed to carry out a task has also been linked to automaticity and the two memory systems would differ in the degree to which they are amenable to automatization, with a higher degree of automaticity characteristic of knowledge harbored by procedural memory (Foerde & Poldrack, 2009; Knowlton, Siegel, & Moody, 2017; Ullman, Earle, Walenski, & Janacsek, 2020). Automaticity is the ability to perform skilled tasks without the need for executive control and is often defined in terms of dual‐task performance: automaticity is achieved when a task can be performed with little or no interference from a demanding secondary task (Poldrack et al., 2005). Studies on language learning see automatic performance as characterized by speed and stability of performance: controlled processes are thought to slow down processing significantly and make it more variable (DeKeyser, 2001; Segalowitz & Segalowitz, 1993; Segalowitz, Segalowitz, & Wood, 1998). Therefore, automaticity is measured as a reduction in the variability of the response time (cf. Segalowitz & Segalowitz, 1993), and this variability is expected to reduce with increased mastery of the language.

Our chosen experimental design can thus be seen as a *dissociation test* where the division of attention, which differentially affects hypothesized long‐term memory constructs, is used to reveal which types of linguistic information each memory system predominantly subserves. Since we test at retrieval stage, we expect the divided attention effect to manifest itself in (an increase in) RT, but not in (a decrease in) accuracy (cf. Craik et al., 1996). Given the massive amount of experience that any healthy speaker will have had with their first language by the time they reach adulthood, we expect all types to be automated in the sense that they can be processed in the presence of a secondary task, while differences in variability may remain. Wherever there are differences, we expect to see a clear dichotomy on a dual‐route model, whereby the lexicon is affected, and syntax is not affected; if morphology is governed by the same principles as syntax, albeit at the word level, then morphology should behave identically to syntax. On a single‐route model where meaning dominates the picture, we expect to see a continuum, whereby the lexicon is most strongly affected, followed by syntax, and tapering off for morphology that conveys rather abstract meaning, if any tangible meaning at all.

The view that memory is composed of distinct systems is based on the idea that there are different types of learning (Knowlton et al., 2017). By way of secondary support, we therefore also run an implicit learning task and an explicit learning task, selecting tasks that engage declarative versus non‐declarative memory as unambiguously as possible.

To measure implicit learning, we ran a probabilistic Serial Reaction Time (SRT) task. The SRT task assesses improvements to immediate memory span for statistically consistent, structured sequences. The SRT task fits the criterion of procedural learning, in that at least a substantial subgroup of participants remain unaware of the underlying sequence, yet still show learning of it through their performance on the task (Willingham, Nissen, & Bullemer, 1989). Like many other tasks used in research on memory, the SRT task has been criticized for its low test–retest reliability (N. Siegelman & Frost, 2015, report test–retest reliabilities of *r* = 0.47 in adults and West, Vadillo, Shanks, & Hulme, 2018 report *r* = 0.21 in children). Nevertheless, it remains the most widely used experimental paradigm to study motor sequence learning (Knowlton et al., 2017; Ullman et al., 2020), and has been used extensively in research on language.

To measure explicit learning ability in the context of language learning, we ran an LLAMA_F task, which is a grammar inferencing test. Llama_F is primarily concerned with the learning of words and agreement features and measures learners’ explicit inductive learning ability, that is, their ability to learn with intention and awareness. As such, it is particularly good at identifying language analytic ability. LLAMA was validated using a 186‐participant sample from three different language backgrounds (English, Spanish, and Chinese) (Granena, 2013). Results yielded acceptable levels of reliability, approaching an internal consistency coefficient of 0.80, as well as showing stability on a test–retest reliability procedure. Principal component analysis showed that the Llama_F task (alongside Llama_B and Llama_E) loaded with cognitive test scores measuring explicit language learning ability, or explicit aptitude.

Overall, if declarative and procedural memory align with explicit and implicit learning respectively, we expect strong explicit learners to excel in the full attention condition but to be affected in the divided attention condition, while strong implicit learners should excel in the divided attention condition.

## 3 Methods

### 3.1 Participants

Considering the typical sample size in studies on memory for language, we recruited 48 participants (nine self‐identified as male and two preferred not to share their gender; mean age = 24.5 years, range 18–62) at the University of Warsaw, Poland. All participants were native Polish speakers and spoke between one and six foreign languages; multilingualism is the norm rather than the exception outside the Anglophone world (Grosjean, 2010). Fifty percent of participants (n = 24) knew two foreign languages, 20.8% (n = 10) knew three, and 14.6% (n = 7) knew four or more, while another 14.6% (n = 7) knew only one. The 91.7% (n = 44) of participants learned English as their first foreign language. The most popular second foreign language was either German or French, and these languages were learned as second foreign language by 27.1% (n = 13) of participants. All participants were in higher education or had already obtained a degree. Roughly half of our participants (n = 25) were high school graduates pursuing a BA. The participants appeared healthy, did not report any reading disabilities or cognitive impairments, and had normal or corrected‐to‐normal vision. All but four participants were right‐handed. Participants’ identities were anonymized, and a unique numeric code was used throughout the analyses.

### 3.2 Materials

We administered a set of three tasks and a background questionnaire. Our main task was a timed grammaticality judgment task in which we tested a range of linguistic phenomena spanning the linguistic cline from morphology, over morpho‐syntax and syntax to lexical semantics in two experimentally manipulated conditions, designed to reveal dependence on the declarative and procedural systems. Two additional tasks aimed to capture our participants’ implicit and explicit pattern learning abilities; these tasks are the SRT task and the Llama_F task, respectively. We provide more details on each of these tasks below.

#### 3.2.1 Background questionnaire

The questionnaire included questions about participants’ age, gender, educational level and years of education, proficiency in other languages and second language use, reading habits, and handedness.

#### 3.2.2 Timed grammaticality judgment task

We implemented a dual‐task paradigm. This paradigm contrasts a full‐attention condition, in which a main task only is executed, with a divided attention condition in which execution of the main task is paired with a concurrent task.

*Stimuli*. All participants heard 192 Polish sentences in total. The stimuli were divided into two sets (Set 1 and Set 2) of 96 sentences each; half of each set were experimental items and half filler items. This ensured that participants were not able to discover which phenomena were the subject of study, nor detect any associations between types of items and their correctness.

Because readers found it very difficult to enunciate stimuli containing errors in a natural fashion, and we did not want to run the risk that participants would pick up on any subtle hesitations caused by these errors, we relied on text‐to‐speech synthesis to create the audio files for our stimuli. The audio files were generated using Google cloud text‐to‐speech services (Google Inc, 2019), using the pl‐PL‐WaveNet‐B voice (for more details, see https://cloud.google.com/text‐to‐speech/). The generited mp3 tracks were split into sentences using Audacity 2.1.2 on Windows (Audacity Team, 2019) and saved as .wav files. The sound duration range was 2,050—5,550 ms.

Each set of 96 sentences contained 48 incorrect and 48 correct items. Although the correct items are correct along all possible dimensions, we made sure that they also contained the elements we manipulated. The other half of the sentences in each set contained errors. Rather than focusing on syntax versus lexicon, as is customary in this line of research, our items contained four different types of structures. We focused on case and aspect (as representatives of nominal vs. verbal morphology), *that*‐subordination (to represent syntax), and collocations (as instances of lexical semantics). Because, to our knowledge, there is no work suggesting that, for example,, aspectual errors would be more or less severe than case errors, and the phenomena under study are not susceptible to variation, we implemented a binary correct/incorrect judgment task rather than a graded one (for a detailed discussion of graded acceptability judgments in linguistics, see Francis, 2022).

Case marks the grammatical function of a nominal element in a sentence (e.g., subject, direct object, indirect object), while aspect marks on verbs how the event they express extends over time (very roughly, with or without reference to the flow of time and the beginning or end of the event). Case and aspect entertain different relations with the semantics of the nouns and verbs on which they are marked: while cases are not typically analyzed in terms of the semantics of their host noun, the lexical approach to aspect is rather dominant (compare here the classes of state, activity, achievement and accomplishment proposed by Vendler, 1957). Subordination is a type of hierarchical clause organization in which one clause depends on the other. Collocations are words that are habitually juxtaposed with a frequency greater than chance (Evert, 2008). The latter two types are typically used to represent grammar and the lexicon, the traditional foci of research on language and memory (M. Siegelman et al., 2019).

To represent **case,** we included stimuli where a noun was used in the incorrect case, for example, (1), where the instrumental *motywacją* is used instead of the accusative *motywacji*:


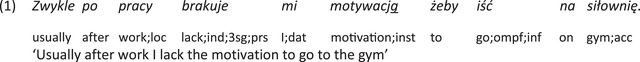


Erroneous sentences for aspect, in which perfective was used instead of imperfective and vice versa, looked like the example in (2), where the imperfective *pić* is used instead of perfective *wypić*.


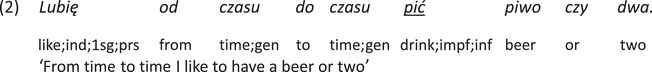


Erroneous **subordination** was exemplified through *że*‐introduced clauses in which a wrong form of the subordinate verb was used, as in 3) where the infinitive is used instead of the past tense, as well as *żeby*‐introduced clauses, as in 4), with the same type of error.


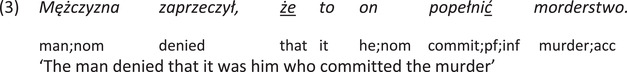



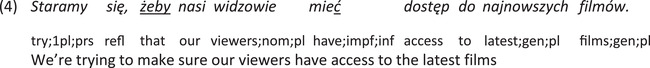


**Collocation** errors, where the word choice was incorrect, were represented by sentences like (5) where *zapis drogowy* is used instead of *przepis drogowy*.


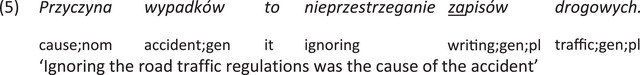


SupMat 1 contains a more detailed explanation of each type and the errors per type. The full stimulus lists (with translation) can be downloaded from https://edata.bham.ac.uk/867/.

*Design*. We implemented a two‐level (single vs. concurrent task condition, ST vs. CT Condition) within‐subject design. The order of conditions (ST or CT) and sentence sets (Set 1, Set 2) was counterbalanced across the participants to remove the potential confounding effects of order or set. For instance, the first participant started with S, Set 1; the second with S, Set 2; the third with C, Set 1 and the fourth with C Set 2. The order of the sentences in each set was randomized, and participants were randomly assigned to a particular experimental setup (i.e., list). The task was implemented using OpenSesame (Mathôt, Schreij, & Theeuwes, 2012).

Each condition started with 10 practice sentences, with half of these sentences containing errors in preposition or number (which were not the type of errors targeted in this study). After sentence presentation participants were given 5,000 ms to press the left arrow to indicate incorrect sentences, and the right arrow to indicate correct sentences. If an answer was not provided within 5000 ms, the next sentence was presented. In the single‐task condition, participants were asked to evaluate whether individually presented sentences were correct, as quickly and accurately as possible. In the concurrent task condition, we employed a preload procedure: participants hold in memory the material for one task while they encode and recall material for the other task. Participants saw a series of three random numbers (ranging from 1 to 9), which they needed to remember and report at the end. Individual numbers were presented visually for 900 ms. Each series of numbers was followed by a sentence and participants were asked to determine whether the sentences were correct, using the same settings as in the baseline condition. After they had provided their correctness judgment, participants were asked to report the three numbers they had seen at the start of the trial.

We set the number of digits to be retained and recalled to three for all participants. This is justified for a number of reasons. First, there is evidence that *differences* in span do not affect sentence processing (see Caplan & Waters, 1999, pp. 80–84 for a review); what counts is the fact that there are concurrent demands. Concurrent memory demands are typical when processing language, and these demands are independent of an individual's WM capacity. Second, studies with a group of L1 Russian speakers that was similar in terms of education and foreign language knowledge had shown that 20% of participants could only hold five digits in memory on the forward digit span task alone, that is, without concurrent language processing load. Mulligan (1997) found that a five‐digit load significantly worsened performance on memory tests. Because our interest is not in understanding WM but in loading it in an ecologically plausible way, we fixed the load at three for all participants. Third, despite the fact that three digits will not have been the max span for some participants, it will have loaded their WM. This assumption is supported by findings from similar groups of participants to whom operation span, reading span, and symmetry span tasks were administered; in their entirety, these tasks resemble the timed grammaticality judgment task we ran here. Medimorec, Mander, and Risko (2018) report OSPAN M = 3.03 for a sample of Canadian undergraduates, while Medimorec, Milin, and Divjak (2021) report OSPAN M = 4.13, RSPAN M = 3.6, and SYMSPAN M = 2.36, and Medimorec, Milin, and Divjak (2020) report RSPAN M = 3.53 for a sample of British undergraduates.

### 3.3 Learning tasks

To measure explicit language learning ability, we ran an LLAMA_F task, which is a grammar inferencing test. To measure implicit sequential pattern learning ability, we used a variant of the multi‐choice, disjunctive SRT task (Vakil, Bloch, & Cohen, 2017).

[1] Measurement of explicit processes: Llama F task

LLAMA_F is a grammar inferencing task and can be downloaded from https://lognostics.co.uk/tools/llama/. Rogers, Meara, Barnett‐Legh, Curry, and Davie (2017) found that all Llama tests are gender and language neutral, and not influenced by experience playing logic puzzles. Formal education qualifications do show a significant advantage on Llama_F, as does prior L2 instruction, but our participant pool is rather homogenous in these respects.

*Stimuli and procedure*. During the presentation phase, a participant is shown a series of pictures depicting shapes and objects, and a short sentence in an artificial language that describes each picture. The task is to figure out how the descriptions relate to the pictures. From this, some words and some grammatical features (i.e., morphological agreement) of the language can be learned. After 5 minutes, participants are presented with a new set of pictures that incorporate new elements. Each picture is accompanied by two sentences and participants have to choose which description is correct. If they have internalized the grammatical rules during the presentation phase, they should be able to select (some) grammatically correct descriptions. Five points are awarded for a correct answer and five points are deducted for an incorrect choice.

*Data pre‐processing*. Scores for the Llama_F test range between 0 and 100 and the Llama manual groups them into four brackets. A score below 15 is considered very poor, and probably due to guessing. A score between 20 and 45 is an average score, and most people are expected to fall in this range. A score between 50 and 65 is a good score, while a score of 75 and above is considered outstanding; few people are expected to achieve the highest score. We grouped the scores of our participants into these same four brackets. Our sample consisted of a large number of analytically strong language learners, with 18 obtaining a score of 75 and above, 11 scoring between 50 and 65, 16 scoring in the average range between 20 and 45, and 3 scoring less than 20. The two participants who scored 0 and the one participant who scored 10 were removed for analysis.

[2] Measurement of implicit processes: SRT task

The multichoice, disjunctive SRT task (Vakil et al., 2017) assesses improvements to immediate memory span for statistically consistent, structured sequences.

*Stimuli and procedure*. The SRT task, administered in one session, took approximately 10 minute to complete, and unfolded as follows. A dot appeared on the screen in one of four positions (Up = 4, Right = 3, Down = 1, Left = 2) and subjects were asked to press the corresponding position on the response pad as quickly as possible. We used second‐order conditional sequences (SOC; Gabriel et al., 2013; Vakil et al., 2017; Wilkinson & Shanks, 2004), meaning that a target location could be predicted only if the two preceding locations were considered. Following Medimorec et al. (2021), we used two sequences: “342312143241” and “341243142132” (adopted from Wilkinson & Shanks, 2004). Each sequence served either as the learning or the interfering sequence, and the order of sequences was counterbalanced across participants. The experiment began with 12 practice trials, consisting of randomly generated sequences. The experiment consisted of six blocks, each containing a 12‐element sequence repeated five times (i.e., 60 trials within a block). The target remained visible until a response key was pressed, triggering another trial. The first four blocks were learning blocks (Block 1–Block 4). Each of these blocks started from a different point in the sequence. The learning blocks were followed by an interfering block, containing a different 12‐element sequence (Block 5). Finally, the original sequence was reintroduced in a recovery block (Block 6). Subjects were not alerted when they moved from one block into the next.

*Explicit awareness questionnaire*. To assess sequence awareness, participants were asked the following questions after they had completed the SRT task: (1) Did you notice anything special about the experiment? (2) Did you notice any patterns during the experiment? (3) If so, could you explicitly recall the pattern? (4) If you think you can recall the pattern, please recreate it now. Out of 48 participants, 17% (n = 8) reported that they noticed something particular about the experiment. In answer to question 2, 41% (n = 20) replied that they noticed a pattern, and 16 people (33% of all respondents) were convinced they could repeat it. The 22 participants who attempted to reproduce the pattern produced sequences ranging from two (eight participants) to four (one participant) correct consecutive positions. The results suggest that while many participants noticed a pattern, they were not able to reliably reproduce it when asked to do so.

*Data pre‐processing*. A density plot of response time latencies revealed the presence of some outliers (both short and long). Retaining only the training blocks, we removed 0.24% from both extremes (28 data points in total) and inversely transformed the remaining latencies to obtain a symmetric, Gaussian‐like distribution; following Baayen and Milin (2010), we applied a −6,000/RT transformation to avoid too narrow a range of transformed latencies and a change in the expected and common directionality of the effect.

We fit a linear mixed effects regression model to participants’ transformed RT latencies to measure their implicit learning aptitude. Intercept and time (trial order) were the main fixed predictors, while the random effects were by‐participant intercept and slope adjustments. Note that our measure of implicit learning thus includes improvements due to both perceptual and motor learning; this is justified because procedural memory supports the learning and execution of both cognitive and motor skills (Ullman, 2004). From the fitted model, we extracted the random time‐slope adjustment to be used as our main measure of individual differences in implicit learning. Scores from the SRT task ranged from −1.4738 to −0.0319 around the main trend line of trial of −0.5854. For statistical modeling, the raw scores were categorized into four quartile groups, as suggested by the histogram; this mirrors the four categories for the LlamaF task. Simple bivariate correlation did not show any concerning overlap between the indicators of implicit and explicit processes (Kendal's τ=0.072;t=0.475;df=43;p>.1).

## 4 Procedure

Testing took place in quiet rooms on the University of Warsaw campus in Poland. Individuals were tested either in groups of two or individually with one experimenter present at all times. Seating arrangement allowed sufficient separation ensuring no interference in any way with the testing procedure. Prior to commencing the experiment participants were provided with an information letter and written consent was obtained from each participant. They were also advised of the possibility to stop or withdraw from the experiment at any time.

All experimental tasks were administered using two identical Lenovo ThinkPad X1 Carbon laptops with an Intel(R)Core(TM) i7‐8565U processor, 16GB of RAM, and a 64‐bit Windows 10 operating system. Participant responses were recorded using wired Apple low latency USB keyboards (A1243). All on‐screen instructions were in Polish. The auditory stimuli were presented to participants through Bose QuietComfort Noise Cancelling QC35 II Over‐Ear Wireless Bluetooth headphones. Two iPads were used to collect questionnaire responses.

Participants, seated in front of the laptop, were asked to focus on grammar and vocabulary, and not to judge the pronunciation (i.e., accent, intonation) of the binaurally presented stimuli. After they had completed the main task, they took the SRT task and the Llama_F task. Demographic questionnaires were administered at the end of the session, except for the last two participants who completed these first. There were no designated breaks except short intervals allowing the experimenter to switch between the tasks. The entire session took approximately 60–70 min. In return for their time, each participant received a monetary compensation of PLN40 or £7.5.

## 5 Results

We used a dual task paradigm, which is known to affect access to declarative and procedural memory differently, to test which dimensions of language knowledge are likely subserved by procedural or implicit memory and which ones depend more on declarative or explicit memory.^2^ Knowledge about the characteristic memory signatures for each of these different types of linguistic information can also be used to arbitrate between dual‐ and single‐route linguistic theories. In this section, we analyze the speed, accuracy, and consistency of the participants’ judgments in the single‐task and concurrent task conditions. All three analyses make it possible to compare performance within and across conditions, allowing us to detect how different types of linguistic knowledge respond to single versus concurrent task demands in terms of speed, accuracy, and consistency of judgment. We also report how different implicit and explicit learning profiles are affected by the two conditions.

### 5.1 Speed of judgment

The analysis of the response latencies is based on 3,661 out of the 4,522 available data points: three participants were excluded as they did not meet the 20‐points threshold for the Llama_F scores (n = 280 or 6.2% of data); we removed n = 310 (6.8%) erroneous datapoints from the timed grammaticality judgment task where the participant judgment did not match the experimenter judgment; and we removed a further n = 271 (6%) datapoints were the three digits were not correctly returned at the end of the trial. This resulted in a total loss of 19% of all datapoints. Note that 57 datapoints had mismatching grammaticality and mismatching digits; 189 datapoints had mismatching grammaticality but matching digits; 602 datapoints combined matching grammaticality with mismatching digits. Aspect and collocations had significantly more missing matching digits than Case and Syntax ($\chi^{2}=\;12.96;df\;=\;3;p\;=\;.005$).

We used the **R** Environment for Statistical Computing (version 4.0.3: R Core Team, 2020) and the **mgcv** package (version 1.8‐33; Wood, 2006, 2011) and fitted an ANCOVA‐like model with four categorical predictors (Type, SRT, Llama_F, and Condition) and one covariate (TrialOrder). Specifically, our analytical efforts focused on the two‐way interactions of Condition (Single Task versus Concurrent Task) with the other three categorical predictors: Type (Aspect, Case, Subordination, Collocation), SRT (with four quartile groups: Slow, Avg. Slow, Avg. Fast, Fast), and Llama_F (grouped into four brackets and retaining the three highest groups, as per the Llama manual: Avg. Low, Avg. High, High). Additionally, we included TrialOrder (scaled) as control covariate (following Baayen & Milin, 2010), and random effects: intercept adjustments for Items, and factorial smooths for TrialOrder by Participant. As the name suggests, random effects are included to account for random variations among Items and Participants. The factorial smooth we included additionally handles the individual random variation over the course of experiment (which can be due to, for example, fatigue, loss of attention, boredom, etc.). The response time latencies (RTs) were log‐transformed to facilitate statistical modeling (cf. Baayen & Milin, 2010).

**Fig. 1:**
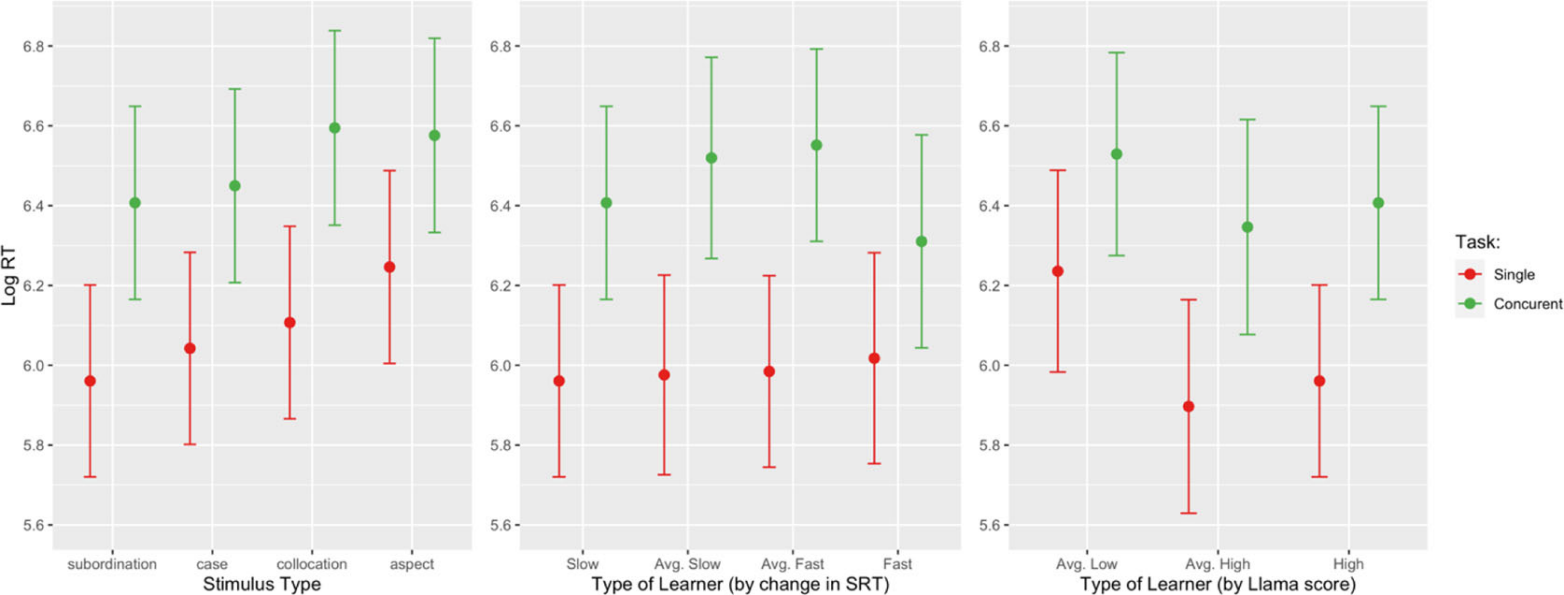
Response latencies for the four stimulus Types across ST and CT conditions (left panel); response latencies for the four types of implicit learners across both Conditions (middle panel); response latencies for the three types of explicit learners across Conditions (right panel). Whiskers represent the 95% lower and upper confidence interval limits.

The final model was tested against several “reduced” models: one without interactions, one with control predictors only, and a null model containing only a constant term (the intercept) and all random effects. The model comparisons were done using chi‐squared tests of AIC (Akaike Information Criterion) scores, as implemented in the **itsadug** package (version 2.4; Van Rij, Wieling, Baayen, & van Rijn, 2020) in **R**. The final model had a significantly better fit than the second‐best one with main effects only ($\chi^{2}=\;41.37;df\;=\;8;p<.0001$). To ensure the robustness of our findings and interpret null‐findings, all models were also run as Bayesian models using the **brms** package (Bürkner, 2017, 2018). The Bayesian results support our final model; the complete summary tables (A, B, C, and D) are given in SupMat 2. Fig. 1 summarizes the findings, and we proceed to discuss specific differences, of theoretical significance for the present study, using Wald's test for comparisons (following Divjak, Milin, & Medimorec, 2020 ).

There was a significant interaction of Condition by Type on RT (F=2.786;df=(1,3);p=.0393). As the left panel of Fig. 1 shows, the difference between the ST versus CT Condition was the most pronounced for Collocation, and then, in decreasing order for Syntax, Case and, finally, Aspect (the respective Chi‐square values are 73.60,67.10,54.82,33,81, with all p<.0001). Within ST, the differences between Aspect and Case ($\chi^{2}=\;4.706;p\;=\;.03$) and between Aspect and Subordination ($\chi^{2}=\;9.236;p\;=\;.002$) are significant. Within CT, only the contrast between Subordination and Collocation reaches marginal significance ($\chi^{2}=\;3.896;p\;=\;.048$).

There was also a significant interaction between Condition and both learning measures (SRT: F=8.268;df=(1,3);p<.0001, and Llama_F: F=5.711;df=(1,2);p=.0033). These interactions are represented on the mid (SRT) and right (LlamaF) panels of Fig. 1. The indicator of implicit learning (the SRT score) shows a practically flat trend line across levels in the ST Condition (i.e., no change) and in the CT Condition none of the pair‐wise differences reaches significance. All implicit learner levels are significantly affected by concurrent task demands (all p<.0001), with strong implicit learners least affected (Single vs. Concurrent $\chi^{2}=\;23.256;p\;=\;.00001$) and significantly less than the other three levels combined ($\chi^{2}=\;31.274;p\;=\;.00001$).

Finally, as depicted in the right panel of Fig. 1, all differences between Conditions for each explicit learner type are highly significant (all p<.0001). While in the ST condition, AverageLow scorers on the LlamaF task are significantly slower than both AverageHigh and High scorers ($\chi^{2}=\;8.85;df\;=\;1;p\;=\;.003$), in the CT condition, they no longer differ significantly in time to decision ($\chi^{2}=\;2.30;df\;=\;1;p>.10$).

### 5.2 Accuracy of judgment

**Fig. 2:**
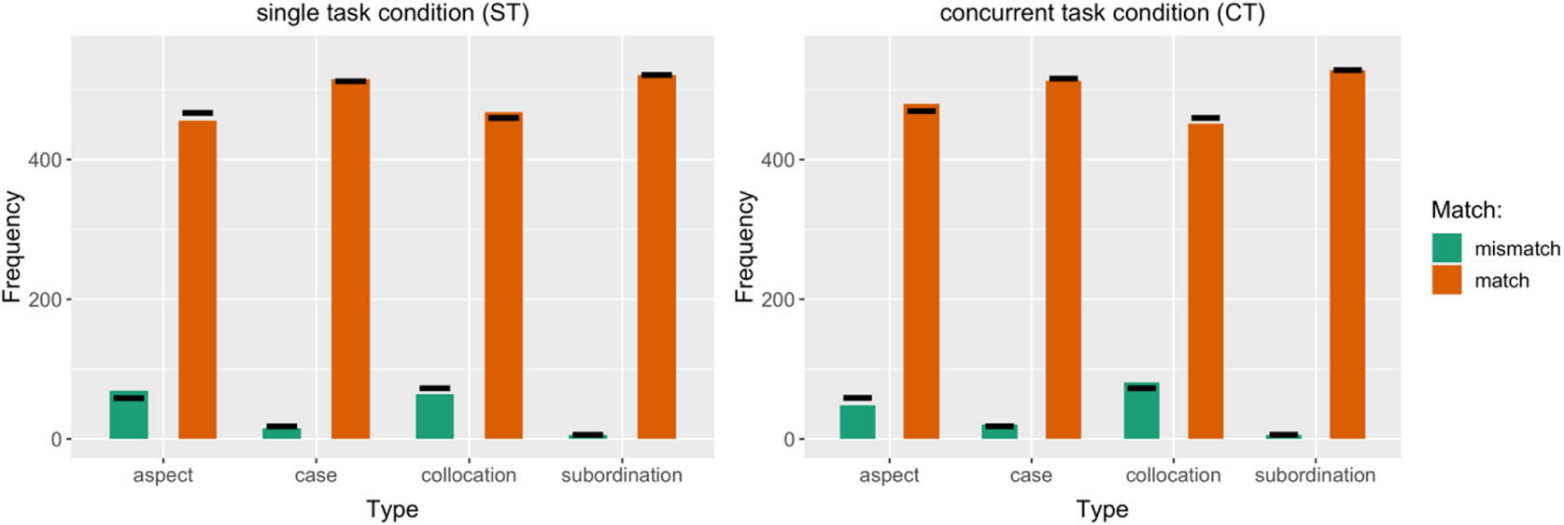
Plot of observed versus predicted frequencies for matched and mismatched responses across two experimental conditions (left and right panels) and four language types. The bars represent the observed frequencies (green for Mismatch, orange for Match) and the black horizontal lines represent the predicted frequencies.

With only 7.3% (*n* = 310) of participants' judgments classed as not matching the experimenter judgment, the accuracy of the responses in our study was very high; we removed a further n = 271 (6%) datapoints where the three digits were not correctly returned at the end of the trial. Because of the high accuracy, there was an imbalance in numbers of items per category (Match = 1 vs. 0, see Fig. 2). For this reason, we relied on log‐linear modeling (LLM, implemented in the core of the **R** software environment) to analyze the accuracy of the responses. LLMs are not constrained by specific distributional assumptions, and are sensitive only to the total number of zero cells and to the number of cells with structural zeroes (details in Rudas, 2018).

We fit a series of LLMs, with the agreement between participant and experimenter judgment (ResponseAccuracy: Match 1 vs. 0) as the dependent variable and Type of linguistic stimulus (Aspect, Case, Subordination, and Collocation) and experimental Condition (Single vs. Concurrent Task) as the main predictors. We also tested the effects of participants’ explicit and implicit learning abilities as captured by the Llama_F task and the SRT task, respectively. These two variables of individual differences, however, did not prove to be predictive of the Match between participants’ and experimenters’ judgments and we removed them from further analyses of participants’ ResponseAccuracy.

The simplest model with a likelihood ratio test statistic that would confirm a good model fit contained only the one direct effect of Type of linguistic stimulus on ResponseAccuracy (Likelihood Ratio = 7. 657; df = 4; *p*‐value = .1050).^3^ A direct effect of Condition on ResponseAccuracy was not statistically justified, as the Likelihood Ratio remained unaffected (i.e., the “improvement” was a mere 1.058) with one additional degree of freedom lost (due to the additional direct effect of Condition; *p* = .7). In other words, in the terminology of LLM fitting, this shows the conditional independence of Match and Condition given the direct effect of Type on Match. The results are summarized in Fig. 2.

The retained Log‐Linear model shows that the chance of encountering a Match between participant and experimenter judgment increases significantly for Subordination and Case, compared to Aspect and Collocation (the LLM multiplicative parameters for interaction between Match and Type are, respectively: 0.7892, 0.1937, −0.4493, −0.5336). However, while the Match rates for the stimulus Types differ, with Subordination and Case causing significantly fewer mismatches than Aspect and Collocation, this relation was not further affected by experimental Condition. Neither was there an interaction of Condition with implicit or explicit learning ability as far as accuracy of judgment is concerned.

### 5.3 Consistency of judgment

**Fig. 3:**
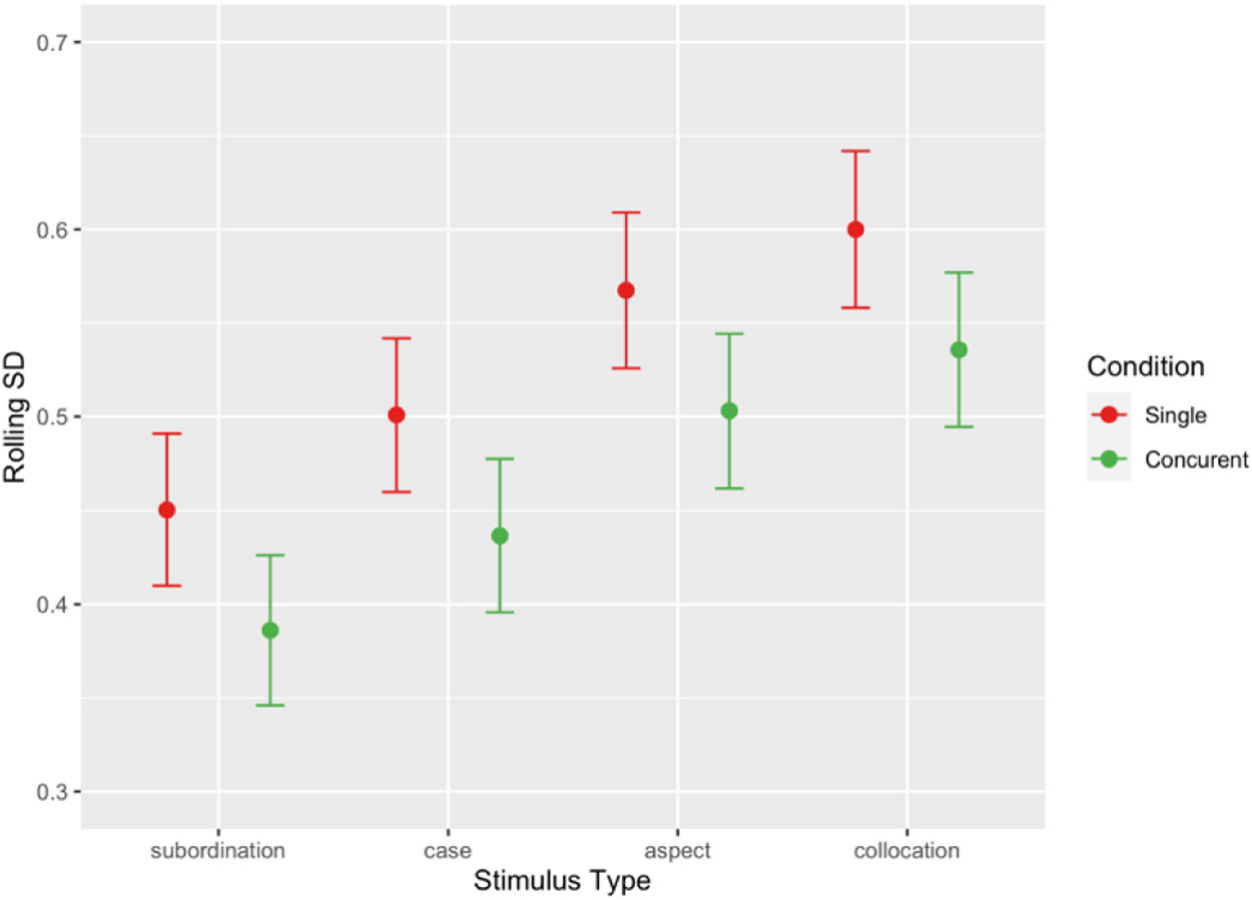
Variability (i.e., moving SDs) for the four stimulus Types across Single task and Concurrent task conditions. Whiskers represent the 95% lower and upper confidence interval limits.

In our final analytic step, we analyzed the dynamic aspects of the participants’ behavior and modeled the *variation* in the time taken to reach a decision *across experimental trials* (in order of presentation) in both ST and CT Conditions and across four grammatical Types (Syntax, Case, Collocation, and Aspect). Following Milin, Divjak, and Baayen (2017) and Divjak and Milin (2020), we used moving (or rolling) standard deviations (SDs); the rolling SD correlates perfectly (*r* = 0.99) with the older coefficient of variation of lexical decision RT (CV_RT_)—the SD of RT divided by mean RT—proposed by Segalowitz and Segalowitz (1993) as a measure of automaticity. These moving SDs were calculated over three consecutive trial latencies, which maximizes the number of available datapoints (moving SDs). We utilized the **qgam** package (Fasiolo, Goude, Nedellec, & Wood, 2021) for **R**, and fitted a quantile generalized additive mixed‐effects model (QGAMM), which is suitable for analyzing moving SD as their residuals cannot be assumed to follow a Gaussian (Normal) distribution (Quantile Regression does not assume any particular form of error term distribution; cf. Koenker, 2005). We evaluated the resulting model at the median (quantile = 0.5), the typical evaluation point (cf. Divjak & Milin, 2020; Schmidtke, Matsuki, & Kuperman, 2017; Tomaschek, Tucker, Fasiolo, & Baayen, 2018). As with the analysis of speed of judgment (i.e., RTs), we confirmed the model against its Bayesian alternative, using the **brms** package (Bürkner, 2017, 2018). The Bayesian analysis, with Asymmetric Laplace link function to allow for quantile modeling, supported our final model, the results of which are condensed in Fig. 3, while the complete summary tables are given in SupMat 2.

We present a simple model with two main fixed factors: Type and Condition.^4^ An analysis of the consistency (or variability) in time to judgement shows that Type remains a highly significant main effect ($\chi^{2}=\;88.760;\;df\;=\;3;p<.0001$), as is Condition ($\chi^{2}=\;37.265;\;df\;=\;1;p<.0001$) but they do not interact significantly (p>.1). In both the ST and the CT Condition, the same two groups emerge in terms of the variability they invoke in time to decision: Syntax and Case, which invoke significantly less variability versus Collocation and Aspect, which invoke significantly more variability (combined contrast: $\chi^{2}=\;29.424;\;df\;=\;1;p<.0001$). In addition to that, all consecutive contrasts are significant except the difference between Aspect and Collocation (Subordination vs. Case: p=.003; Case vs. Aspect: p=.0002; Aspect vs. Collocation: p>.05).

### 5.4 Summary of findings

Table 1 summarizes the results of the interaction between Type and Condition. Significant effects are marked with a **✓** and the χ^2^ is given between brackets.

  

**Table 1:** Summary of significant results for the interaction between Type and Condition

		**Speed**	**Accuracy**	**Consistency**
**GRAMMAR**	**Aspect**	$\chi^{2}=\;33,81,\;p<.0001$	n.s.	n.s.
	**Case**	$\chi^{2}=\;54.82,\;p<.0001$	n.s.	n.s.
	**Subordination**	$\chi^{2}=\;67.10,\;p<.0001$	n.s.	n.s.
**LEXICON**	**Collocation**	$\chi^{2}=\;73.60,\;p<.0001$	n.s.	n.s.

Table 2 summarizes the results within Condition. For significant differences, the χ^2^ is given. Because for the Speed of Judgment analysis, Type and Condition interact, the differences between Types are different in the Single versus Concurrent conditions; thus, the values are given in two rows, with the Single Task on the first row and the Concurrent Task on the second row, per cell. For Accuracy, given the independence of Condition and Match, χ^2^ comparisons are calculated between the average frequency per Type (against equiprobable frequency, i.e., independence). For Consistency, specific contrast values are identical in both Conditions, given the independent effects of Type and Condition.

**Table 2:** Summary of significant results within Conditions, including marginally significant contrasts (.01 < *p* < .1) between square brackets

		**Case**	**Subordination**	**Collocation**
**Speed**	**Aspect**	$\chi^{2}=\;4.706,\;p\;=\;.03$ n.s.	$\chi^{2}=\;9.236,\;p\;=\;.002$ $\left[\chi^{2}=\;3.192,\;p\;=\;.07\right]$	$\chi^{2}=\;4.706,\;p\;=\;.03$ n.s.
	**Case**	n.s.	n.s. n.s.	n.s. n.s.
	**Subordination**	n.s.	n.s.	$\chi^{2}=\;4.706,\;p\;=\;.03$ n.s.
**Accuracy**	**Aspect**	n.s.	$[\chi^{2}=\;3.216,\;p\;=\;.07$]	n.s.
	**Case**	n.s.	n.s.	$\left[\chi^{2}=\;3.051,\;p\;=\;.08\right]$
	**Subordination**	n.s.	n.s.	$\chi^{2}=\;4.294,\;p\;=\;.04$
**Consistency**	**Aspect**	$\chi^{2}=\;14.20,\;p<.0001$	$\chi^{2}=\;45.59,\;p<.0001$	$\left[\chi^{2}=\;3.289,\;p\;=\;.07\right]$
	**Case**	n.s.	$\chi^{2}=\;8.751,\;p\;=\;.003$	$\chi^{2}=\;31.09,\;p<.001$
	**Subordination**	n.s.	n.s.	$\chi^{2}=\;73.54,\;p<.0001$

## 6 Discussion

We set out to determine the behavioral signatures of memories for different *types* of linguistic knowledge in a population of healthy adult L1 speakers. To this end, we defined a cline of linguistic types, from morphology (case and aspect) over syntax (subordination) to lexical semantics (collocation) in Polish, a Slavic language much richer in form variation than English. Participants were asked to judge sentences containing correct and incorrect instances of case, aspect, subordination, and collocations under dual‐task conditions: a main condition in which only the grammaticality of stimuli was assessed and a condition in which grammaticality judgments were given while a digit span task was performed. This yielded three types of measures for further analysis, that is, judgment response time latencies (RTs), judgment accuracy (match/mismatch between participant and experimenter judgment), and judgment consistency (moving SDs over consecutive RTs).

Recall that, for judgment speed, there was a significant interaction of Condition and Type whereby all Types were affected by the memory load, albeit to different extents: in order of magnitude, collocations were followed closely by subordination, which was followed by case and then aspect. In the single‐task condition, case and subordination group together, as do aspect and collocations. This pattern also shows under concurrent task conditions, but it is a consequence of collocations being affected most and aspect least by memory load. For accuracy, there was no effect of Condition with case and subordination consistently causing significantly fewer mismatches between participant and experimenter judgment than aspect and collocation. Analysis of the rolling SD on the time taken to reach a decision showed the same pattern: there is no interaction between Type and Condition and instead, case and subordination consistently show less variation in time to decision. We will discuss the implications of our findings for theories of language and for models of memory for language in more detail.

### 6.1 Memory signatures for language structures and learning abilities

The idea that there are different types of learning (Knowlton et al., 2017) closely matches the view that memory is composed of distinct systems. It is generally assumed that declarative memory supports explicit learning, while procedural memory is specialized for implicit learning (although declarative memory has capacity for implicit learning, too). Given the parallel (but not perfect overlap) between memory structures (declarative vs. procedural) and types of learning (explicit vs. implicit), we observed an interaction between Condition and both learning measures in terms of speed of judgment, in the expected direction. Strong explicit learners rely heavily on declarative memory: they benefit most from having WM available because it facilitates access to declarative memory but suffer significantly more from concurrent task demands because WM load impedes access to declarative memory. Our findings do support such conclusion as strong explicit learners appear to be significantly faster in the single‐task condition and more affected by concurrent task demands than weak explicit learners. On the other hand, strong implicit learners rely heavily on procedural memory. Implicit learners did not differ significantly from each other in the single‐task condition, but each of the four types of implicit learners were significantly slower in the concurrent task condition, with the fast learners least affected. Fast learners outperform all others in the concurrent task condition that impedes access to declarative memory but leaves access to the procedural system unobstructed.

### 6.2 Memory signatures for language structures: Morphology, syntax, and the lexicon

Our lexical structures, collocations, are common word combinations, that is, words or phrases that are typically used together but their mutual preference might not be expected from their meaning. They are examples of declarative memory *par excellence*: declarative memory was constructed to harbor these idiosyncratic structures (see insights from experimental psychology, e.g., McKee & Squire, 1993, from an evolutionary perspective, e.g., Manns & Eichenbaum, 2006, as well as from neurobiological mappings, e.g., Eichenbaum, 1997; Javadi & Walsh, 2012). The behavioral memory signatures found while judging collocations should be found in other types of linguistic structures that are handled by declarative memory as well. Collocations clearly show behavior that is consistent with access to the information being slow and controlled (MacDonald, 2008; Richmond & Nelson, 2007), as well as fallible (Squire et al., 1993). Judgment times are long in single‐task condition already and lengthened further significantly under concurrent task conditions. Mismatches between participant and experimenter judgment are consistently significantly higher than for case and subordination. There is also significantly more variability in lexical judgments than for case and subordination in the single‐task condition; this variability remains high under concurrent task demands.

Despite the fact that syntax has traditionally been used as counterpart of the lexicon, the findings for subordination are inconsistent with that claim: the assumption that syntax should be taken to be a prototypical representative of procedural knowledge, where access to information is fast and automatic as well as reliable (MacDonald, 2008; Squire et al., 1993), does not receive strong support. The present results show that judgment times are short in the single‐task condition but are significantly lengthened under concurrent task demands. In fact, under concurrent task demands, syntax does not differ significantly from the lexicon. The observation that syntax does not clearly appear as harbored by procedural memory is in line with findings from a meta‐analysis of neuroimaging studies (fMRI or functional Magnetic Resonance Imaging and PET or Positron Emission Tomography) on syntactic processing (Walenski, Europa, Caplan, & Thompson, 2019). Yet, there are also differences between syntax and the lexicon: regardless of condition, subordination is judged more accurately than collocations, while variability in time to decision is lower for syntax (i.e., consistency in decision‐making is higher). Across all measures, processing subordination appears to pose demands on memory that are dissimilar to the demands that lexical items pose, yet syntax is affected by dual task demands virtually to the same extent as the lexicon.^5^

The remaining two Types likewise show traces of procedural memory, albeit in different ways and to different extents. Aspect shows an interesting pattern across tasks and conditions, and one that is the opposite of what we obtained for syntax. For speed of decision, in the single‐task condition, aspect groups with collocations and requires the longest time to decision; this pattern is also observed in the concurrent task condition. Across Conditions, however, aspect is affected least of all Types by dual task demands. For accuracy, there was no effect of Condition, with both aspect and collocation consistently causing significantly more mismatches than the other Types; recall also that the data for aspect and collocation contained significantly more non‐matching digits. Likewise, in terms of overall variability in time to decision, aspect pairs with collocations within Conditions, and variability is not affected by memory load. Across all measures, processing aspect appears to pose demands on memory that resemble more the demands that lexical items pose than the demands that syntax poses. Yet, aspect is least affected by dual task demands. Comparing the ERP signatures for morpho‐syntactic and semantic violations with those obtained for aspectual violations, Flecken, Walbert, and Dijkstra (2015) likewise found that processing aspectual violations did not show any of the known ERP effects. They conclude that aspect processing reflects operations that are neither purely semantic nor exclusively morpho‐syntactic in nature.

For case, decision times are short in the single‐task condition but are significantly lengthened by concurrent task demands, although to a lesser extent than collocation and subordination. Compared to collocation, mismatches between participant and experimenter judgment are significantly lower for case, regardless of Condition. Variability in time to decision is low under both task conditions and is not significantly affected by concurrent task demands. Looking across all measures, of all types, case comes closest to being under thexclusive purview of procedural memory. This behavioral signature, or a more extreme version, should therefore be found in other types of linguistic structures that are handled by procedural memory. Using fMRI, Newman, Supalla, Hauser, Newport, and Bavelier (2010) found evidence of the existence of distinct neural mechanisms for processing specific types of grammatical structures; they, too, observed that inflectional morphology appeared to mobilize brain areas typically associated with procedural memory. Likewise, Ullman (2016) reports that morphemes, which are not clearly linked to conceptual meaning but are instead tied to grammatical structure, are linked to areas that support procedural learning and memory, rather than declarative memory.

These findings highlight that the differences between types need to be taken into account when using language stimuli for the study of memory. The crisp divide between declarative and non‐declarative memory domains, conveniently mirrored in the divide between lexicon and grammar, was a truly appealing proposal that has dominated decades of theorizing and research across the cognitive (neuro‐)sciences (for an overview, see M. Siegelman et al., 2019). As empirical evidence accrues, however, a new picture is starting to emerge, which reveals that the two memory domains overlap structurally in the brain, and jointly participate in various memory functions. Findings based on syntax may not be representative for any other types that exhibit patterned activity that is typically classed as “grammar.” Furthermore, there may well be differences between members of the same linguistic subcategory: both case and aspect are traditionally considered as morphology, but they behave in very different ways. The results we present thus also challenge a model that highlights the overlap of the two memory domains, in that some linguistic phenomena seem to bank on this overlap more than other phenomena. This should be taken into account when formulating theories of memory and learning and designing studies to test them, but also when selecting linguistic types for assessing memory in clinical populations (Varkanitsa & Caplan, 2018).

The split between lexicon and grammar also fit the long dominant generative approach to language with its focus on English. Work on language memory is now being challenged by growing concerns that research on language cannot be the *science of English*: English is exceptional in its formal simplicity. Many other languages offer an exciting richness that disobeys the strict grammar versus lexicon divide. Naturally, including such languages and their unique complexities is desirable: it is likely to change how we conceptualize memory for language, and hence how we design behavioral and neuroimaging studies and interpret the data they produce. Future work might also want to consider including task specifics (e.g., online processing, offline metalinguistic judging) as an additional experimental layer as it may impact the nature of the cognitive processes the participants engage in.

### 6.3 Memory signatures for language structures and theories of language cognition

Our results are not fully predicted by any one theory of language cognition; instead, both dominant frameworks predict the results only partially. In line with what would have been expected on a dual‐route, generative approach, some linguistic types do appear to be under the purview of declarative memory: for the lexicon (collocations), access to declarative memory is/remains crucial, even in a highly educated population of healthy L1 speakers. These findings go against blanket claims that, with exposure and proficiency, the procedural system takes precedence in supporting language processing (Opitz & Friederici, 2003; Ullman & Lovelett, 2018); clearly, this relation is modulated by the nature of the type of linguistic unit that is being processed.

However, while our behavioral memory signatures confirm the division between clearly declarative and more procedural language abstractions, they also suggest that the dividing line, if any, falls in a different place than assumed on a dual‐route, generative approach: analysis of response latencies under single and concurrent task conditions revealed that, while memory load had differential effects on the four linguistic structures, syntax (subordination) did not differ significantly from the lexicon (collocations) in this respect. The counterpart of the lexicon is not syntax (subordination), but morphology: it is aspect that displays the hallmark features of procedural memory under memory load.

Given the (variable) traces of declarative memory across Types, our findings do not mesh either with strong usage‐based claims that all linguistic knowledge is represented in the same format, as pairings between forms and their meaning, and therefore, depend on the same learning mechanisms and rely on the same memory systems (Llompart & Dabrowska, 2020). Our study shows that while it may be so that grammar also carries meaning, some form‐meaning pairings are privileged over others: those forms that constitute lexical items point to meanings that differ qualitatively from the meanings activated by forms that are traditionally considered grammatical and are handled, at least to a considerable degree, by different memory systems. Furthermore, we found that the different Types show traces of procedural memory in different ways and to different extents. This idea of a cline, from more grammatical to more lexical meanings, does fit well with single‐route usage‐based approaches where meaning dominates the picture. A continuum is expected, whereby the lexicon is most strongly “affected,” and this effect tapers off for morphology and syntax that convey rather abstract meanings, if any tangible meaning at all. That the language processing space appears as graded rather than categorical corroborates our current understanding of how human memory works and how it is embedded in the brain: it is rather a case of collaboration than of cohabitation.

A cline also emerges in terms of automatization. Automatization, and degrees of automatization in particular, play a differential role in memory systems, with a higher degree of automaticity characteristic of knowledge harbored by procedural memory. The degree of automaticity has been defined, generally, as the reduction in cost the secondary task has on the performance of the main task, which would manifest itself as a reduction in the increase of response time (cf. Poldrack et al., 2005), and within studies on language learning, as a reduction in the variability of the response time (cf. Segalowitz & Segalowitz, 1993). We measured automatization as the amount of variability in time to judgment. Likely because of the massive amount of experience participants have with their first language, we did not observe a differential reduction in the cost the secondary task has on the performance of the main task, but we did register a differential reduction in the variability of the response time regardless of condition. The observed, within‐condition type‐related differences in stability of judgment thus point toward different degrees of automatization: syntactic subordination is more automated than morphological case, which is more automated than morphological aspect, which aligns with lexical semantics (collocations). Our findings thus suggest that there would be a cline, from easily automated phenomena to difficult to automate phenomena, not a binary division. However, the within‐condition sequence of types differs from what we found across conditions: within conditions, lexicon and syntax do occupy opposing extremes as a dual‐route model would predict. On a usage‐based approach, it is generally assumed that experience has a differential effect on processing; this has standardly been thought to affect (lexical) tokens, not (grammatical) types, however. Our findings change this.

Overall, across all three analyses and within conditions, morphology (case) and syntax (subordination) pair up and contrast with morphology (aspect) and the lexicon (collocations). Analysis of the RT data within conditions showed that aspect and collocations take longer to judge than morphology (case) and syntax (subordination). Analysis of response accuracy data showed that there is an effect of type on accuracy regardless of condition: morphology (case) and syntax (subordination) are more likely to trigger a matching agreement between participant and experimenter than morphology (aspect) and collocations. Variability analysis revealed a similar pattern with type affecting variability regardless of condition, and again, it is morphology (case) and syntax (subordination) that trigger less variation in time to decision than morphology (aspect) and collocations. Taken together, the findings relating to Speed and Consistency (automatization) reveal a trade‐off between average judgment time and judgment variability, with more time and less variation in the concurrent task (compare Figs. 1 and 3). The observation that, within Conditions, aspect aligns with collocations goes against much work in the generative framework that has traditionally aimed to ascribe as much as possible of the lexicon to syntax, by positing a generative‐like engine for the lexicon, which essentially proposes syntax‐like operations for word formation. It is also routine in much generative literature to use syntactic operations to introduce grammatical aspectual operators (John Beavers, personal communication). This same observation also confirms that the validity of the so‐called lexical approaches to aspect (pioneered by Vendler, 1957 for English and recently adopted by Croft, 2012 but preceded by Maslov, 1948 for Russian). Lexical approaches to aspect assume that aspectual usage is governed largely by lexical factors, where the meaning of a verb implicitly constrains its usage. In other words, on a lexical approach to aspect, the perfective and imperfective aspects do not possess an invariant meaning that is primordial and permeates all of their uses, as assumed by proponents of grammatical approaches to aspect. Instead, the type of action expressed by the verb determines the meaning of the aspectual opposition and explains and predicts aspectual usage. It is this lexical dimension that gives rise to the highly variable and idiosyncratic behavior of aspect.

## 7 Conclusions

Although there is a broad consensus that both the procedural and declarative memory systems play a crucial role in language learning, use, and knowledge, the mapping between linguistic types and memory structures appears rigid and remains underspecified. The binary lexicon‐grammar split has long gone unchallenged, its lure likely strengthened by the focus of generative linguistic theories on these two types of structures and the focus of memory research on a formally simple language such as English that obscures the interdependence of grammar and lexicon. Our findings suggest that the default dual‐route mapping of language systems to memory systems, with declarative memory handling the idiosyncratic lexicon and procedural memory handling the rule‐governed syntactic component, may not accurately reflect the memory demands that processing language poses on healthy L1 users.

The dual‐task paradigm revealed that, of our four linguistic types, lexical collocations are indeed, mainly declarative in nature, while the three other types (aspect, case and subordination) show traces of procedural memory to different extents. Crucially, syntax (subordination) differs least from the lexicon under memory load conditions and the real “opposition” under memory load is one between lexicon and morphology (aspect). Within conditions, however, morphology (case) and syntax (subordination) pair together and differ from morphology (aspect) and the lexicon (collocations), in terms of judgment speed, accuracy, and stability.

Our findings thus confirm both usage‐based and generative views that there is a division between lexicon and grammar, but the division falls in a different place than assumed, and the distinction is graded: the hypothesized grammar (rule)—lexicon (idiosyncrasy) opposition appears as a continuum on which linguistic abstractions can be placed as being more or less “ruly” or “defiant,” and more or less amenable to automatization. This move away from a simple dichotomy fundamentally changes how we think about memory for language, and hence how we design and interpret behavioral and neuroimaging studies that probe into the nature of language cognition.

## Funding

This research was supported by The Leverhulme Trust RL‐2016‐001 to Dagmar Divjak, which funded all authors.

## Conflict of interest

The authors declare that they have no conflict of interest.

## Ethical approval

All procedures performed were in accordance with the ethical standards of the institutional research committee and with the 1964 Helsinki declaration and its later amendments or comparable ethical standards. The study was approved by the University of Birmingham Ethics Committee.

## Informed consent

Informed consent was obtained from all individual participants included in the study.

## Availability of data

The data set supporting the conclusions of this article and the R code necessary to reproduce the statistical models are available as follows:

Stimuli and data: https://edata.bham.ac.uk/867/

R code: https://github.com/ooominds/Memory_Resources_for_Language

## Supporting information



Supplementary information

**Table A**. Generalized Additive Mixed Model fitted to the grammaticality judgment decision latencies (log‐transformed).
**Table B**. Bayesian Generalized Additive Mixed Model fitted to the grammaticality judgment decision latencies (log‐transformed), using 4 chains with 4000 iterations each.
**Table C**. Additive Quantile Mixed Model fitted to the rolling standard deviations over the grammaticality judgment decision times.
**Table D**. Bayesian Additive Quantile Mixed Model fitted to the rolling standard deviations over the grammaticality judgment decision times, using 4 chains with 4000 iterations each.

Supplementary information

## Notes

1 Note that non‐declarative memory was known as procedural memory until Squire (2004), who extended this type of memory to include conditioning, habituation and sensitization, and priming, and renamed it accordingly as “non‐declarative” memory.

2 In this section, capitalized nouns refer to a manipulation level in our study and a variable in our statistical model while non‐capitalized counterparts signal the general referential use of the same term.

3 Note that a favorable model will be indicated by an insignificant *p*‐value between the (formal) prediction and the data. The goal of modeling is, thus, to find the most parsimonious model that is simultaneously tightly fit to the data.

4 There is, however, a more complex model including two 2‐way interactions of Condition with measures of explicit and implicit learning: Condition by Llama_F and Condition by SRT. This model shows a better fit (with the moderate difference of 37 AIC units). After careful inspection of contrasts, we concluded that the improvement is driven by three specific second‐order differences: (a) Llama_F(Avg.Low) : Condition(Single) versus Llama_F(Avg.Low) : Condition(Concurrent); (b) SRT(Avg.Slow) : Condition(Single) versus SRT(Avg.Fast) : Condition(Concurrent); (c) SRT(Avg.Fast) : Condition(Single) versus SRT(Avg.Fast) : Condition(Concurrent). As Bayesian (non‐linear) hypothesis testing, implemented in the **brms** package for **R**, allows a more fine‐grained analysis, we used this to explore the nature and the strength of these effects further. First, these effects reveal that some specific levels of explicit (Llama_F Avg.Low) and implicit (SRT Avg.Fast) learning abilities are more affected by the concurrent task manipulation. They are significant at the *p* = .05 level. At the more conservative level of *p* = .01, the third difference (SRT Avg.Fast between Single and Concurrent conditions) is non‐significant, and at the even more conservative level of *p* = .001, none of the three effects remain significant. For all these reasons taken together, we decided to refrain from discussing the more complex model and its specific differences further.

5 These findings may shed light on a challenging area: equivocal results have been reported in studies probing working memory, which rely heavily on syntactic stimuli. Gordon, Hendrick, and Levine (2002) asked participants to remember a list of nouns while they listened to syntactically simple and complex sentences. The linguistic items in working memory caused interference with complex sentence processing, especially if the words that had to be held in memory were similar to those used in the sentence. Waters, Caplan, and Yampolsky (2003) asked university students to listen to syntactically simple and complex sentences and judge those while performing a digit span task. The digit span task affected sentence processing, but it did so regardless of sentence complexity. Our findings are in line with Waters et al. (2003), in that even relatively simple subordination patterns showed an effect of concurrent task demands, but they also show that syntax behaves in a rather peculiar way.

## References

[cogs13206-bib-0001] Ambridge, B. , & Lieven, E. V. M. (2011). Child language acquisition: Contrasting theoretical approaches. Cambridge: Cambridge University Press.

[cogs13206-bib-0002] Baayen, R. H. , Dijkstra, T. , & Schreuder, R. (1997). Singulars and plurals in Dutch: Evidence for a parallel dual‐route model. Journal of Memory and Language, 37(1), 94–117.

[cogs13206-bib-0003] Baayen, R. H. , & Milin, P. (2010). Analyzing reaction times. International Journal of Psychological Research, 3(2), 12–28.

[cogs13206-bib-0004] Baddeley, A. D. (1986). Working memory. Oxford: Clarendon.

[cogs13206-bib-0005] Baddeley, A. D. (2003). Working memory: Looking back and looking forward. Nature Reviews Neuroscience, 4(10), 829–839. 14523382 10.1038/nrn1201

[cogs13206-bib-0006] Baddeley, A. D. , Lewis, V. , Eldridge, M. , & Thomson, N. (1984). Attention and retrieval from long‐term memory. Journal of Experimental Psychology: General, 113(4), 518–540. 10.1037/0096-3445.113.4.518

[cogs13206-bib-0007] Blevins, J. P. (2016). Word and paradigm morphology. Oxford: Oxford University Press.

[cogs13206-bib-0008] Blumenfeld, R. S. , & Ranganath, C. (2007). Prefrontal cortex and long‐term memory encoding: An integrative review of findings from neuropsychology and neuroimaging. The Neuroscientist, 13, 280–291. 17519370 10.1177/1073858407299290

[cogs13206-bib-0009] Booij, G. (2010). Construction morphology. Language and Linguistics Compass, 4(7), 543–555.

[cogs13206-bib-1001] Brewer, J. B. , Zhao, M. , Marling, T. S. , Desmond, J. E. , Glover, G. H. , & Gabrieli, J. D. E. (1998, March). Event‐related FMRI of medial lobe involvement in declarative memory formation. In Journal of Cognitive Neuroscience, (Vol. 10, pp. 121–121. Cambridge, MA: MIT Press.

[cogs13206-bib-0010] Bürkner, P.‐C. (2017). brms: An R package for Bayesian multilevel models using Stan. Journal of Statistical Software, 80(1), 1–28. 10.18637/jss.v080.i01

[cogs13206-bib-0011] Bürkner, P.‐C. (2018). Advanced Bayesian multilevel modeling with the R package brms. The R Journal, 10(1), 395–411. 10.32614/RJ-2018-017

[cogs13206-bib-0012] Bybee, J. L. (2006). From usage to grammar: The mind's response to repetition. Language, 82(4), 711–733.

[cogs13206-bib-0013] Cabeza, R. , & Moscovitch, M. (2013). Memory systems, processing modes, and components: Functional neuroimaging evidence. Perspectives on Psychological Science, 8(1), 49–55. 24163702 10.1177/1745691612469033 PMC3806137

[cogs13206-bib-0014] Caplan, D. , & Waters, G. S. (1990). Short‐term memory and language comprehension: A critical review of the neuropsychological literature. In G. Vallar & T. Shallice (Eds.), Neuropsychological impairments of short‐term memory (pp. 337–389). Cambridge: Cambridge University Press.

[cogs13206-bib-0015] Caplan, D. , & Waters, G. S. (1999). Verbal working memory and sentence comprehension. Behavioral and Brain Sciences, 22(1), 77–94. 11301522 10.1017/s0140525x99001788

[cogs13206-bib-0016] Chao, L. L. , Haxby, J. V. , & Martin, A. (1999). Attribute‐based neural substrates in temporal cortex for perceiving and knowing about objects. Nature Neuroscience, 2(10), 913–919. 10491613 10.1038/13217

[cogs13206-bib-0017] Chomsky, N. (1965). Aspects of the theory of syntax. Cambridge, MA: MIT Press.

[cogs13206-bib-0018] Chomsky, N. (1995). The minimalist program. Cambridge, MA: MIT Press.

[cogs13206-bib-0019] Cohen, N. J. , & Squire, L. R. (1980). Preserved learning and retention of pattern analyzing skill in amnesia: Dissociation of knowing how and knowing that. Science, 210, 207–210. 7414331 10.1126/science.7414331

[cogs13206-bib-0020] Coltheart, M. (1985). Cognitive neuropsychology and the study of reading. In M. I. Posner & O. S. M. Martin (Eds.), Attention and performance (Vol. 11, pp. 3–37). Hillsdale: Erlbaum.

[cogs13206-bib-0021] Coltheart, M. , & Rastle, K. (1994). Serial processing in reading aloud: Evidence for dual‐route models of reading. Journal of Experimental Psychology: Human Perception and Performance, 20(6), 1197–1211. 10.1037//0096-1523.26.3.1232 10884020

[cogs13206-bib-0022] Coltheart, M. , Rastle, K. , Perry, C. , Langdon, R. , & Ziegler, J. (2001). DRC: A dual route cascaded model of visual word recognition and reading aloud. Psychological Review, 108(1), 204–256. 11212628 10.1037/0033-295x.108.1.204

[cogs13206-bib-0023] Conway, A. R. A. , Kane, M. J. , Bunting, M. F. , Hambrick, D. Z. , Wilhelm, O. , & Engle, R. W. (2005). Working memory span tasks: A methodological review and user's guide. Psychonomic Bulletin & Review, 12, 769–786. doi: 10.3758/BF03196772 16523997

[cogs13206-bib-0024] Craik, F. I. M. , Govoni, R. , Naveh‐Benjamin, M. , & Anderson, N. D. (1996). The effects of divided attention on encoding and retrieval processes in human memory. Journal of Experimental Psychology: General, 125(2), 159–180. 10.1037/0096-3445.125.2.159 8683192

[cogs13206-bib-0025] Croft, W. (2012). Verbs. Aspect and causal structure. Oxford: Oxford University Press.

[cogs13206-bib-0026] Dapretto, M. , & Bookheimer, S. Y. (1999). Form and content: Dissociating syntax and semantics in sentence comprehension. Neuron, 24, 427–432. 10571235 10.1016/s0896-6273(00)80855-7

[cogs13206-bib-0027] Dayan, P. (2007). Bilinearity, rules, and prefrontal cortex. Frontiers in Computational Neuroscience, *1*, 1. 18946523 10.3389/neuro.10.001.2007 PMC2525936

[cogs13206-bib-0028] DeKeyser, R. M. (2001). Automaticity and automatization. In P. Robinson (Ed.), Cognition and second language instruction (pp. 125–151). Cambridge: Cambridge University Press.

[cogs13206-bib-0029] di Sciullo, A. M. , & Williams, E. (1987). On the definition of word. Cambridge, MA: Massachusetts Institute of Technology Press.

[cogs13206-bib-0030] Divjak, D. (2019). Frequency in language: Memory, attention and learning. Cambridge: Cambridge University Press.

[cogs13206-bib-0031] Divjak, D. , & Milin, P. (2020). Exploring and exploiting uncertainty: Statistical learning ability affects how we learn to process language along multiple dimensions of experience. Cognitive Science, 44(5), 32. 10.1111/cogs.12835 32342542

[cogs13206-bib-0032] Divjak, D. , Milin, P. , & Medimorec, S. (2020). Construal in language: A visual‐world approach to the effects of linguistic alternations on event perception and conception. Cognitive Linguistics, 31(1), 37–72.

[cogs13206-bib-0033] Doherty, J. M. , Belletier, C. , Rhodes, S. , Jaroslawska, A. J. , Barrouillet, P. , Camos, V. , … Logie, R. (2019). Dual‐task costs in working memory: An adversarial collaboration. Journal of Experimental Psychology: Learning, Memory, and Cognition, 45(9), 1529–1551. doi: 10.1037/xlm0000668 30407025 PMC6727883

[cogs13206-bib-0034] Eichenbaum, H. (1997). Declarative memory: Insights from cognitive neurobiology. Annual Review of Psychology, 48(1), 547–572. 10.1146/annurev.psych.48.1.547 9046568

[cogs13206-bib-0035] Eichenbaum, H. (2004). Hippocampus: Cognitive processes and neural representations that underlie declarative memory. Neuron, 44(1), 109–120. doi: 10.1016/j.neuron.2004.08.028 15450164

[cogs13206-bib-0036] Ellis, N. C. (2008). Implicit and explicit knowledge about language. In J. Cenoz & N. H. Hornberger (Eds.), Encyclopedia of language and education (2nd ed., Vol. 6, pp. 119–132). New York: Springer.

[cogs13206-bib-0037] Evert, S. (2008). Corpora and collocations. In A. Lüdeling & M. Kytö (Eds.), Corpus linguistics: An international handbook (Vol. 29.2, pp. 1212–1248). Berlin: Mouton De Gruyter.

[cogs13206-bib-0038] Fasiolo, M. , Goude, Y. , Nedellec, R. , & Wood, S. N. J. A. P. A. (2021). Fast calibrated additive quantile regression. *Journal of the American Statistical Association*, *116*, 1402.

[cogs13206-bib-0039] Fedorenko, E. , Behr, M. K. , & Kanwisher, N. (2011). Functional specificity for high‐level linguistic processing in the human brain. PNAS, 108(39). 10.1073/pnas.1112937108 PMC3182706 21885736

[cogs13206-bib-0040] Fedorenko, E. , Blank, I. , Siegelman, M. , & Mineroff, Z. (2020). Lack of selectivity for syntax relative to word meanings throughout the language network. Cognition 203, 104348. 32569894 10.1016/j.cognition.2020.104348 PMC7483589

[cogs13206-bib-0041] Fernandes, M. A. , & Moscovitch, M. (2002). Factors modulating the effect of divided attention during retrieval of words. Memory & Cognition, 30(5), 731–744. 10.3758/BF03196429 12219890

[cogs13206-bib-0042] Flecken, M. , Walbert, K. , & Dijkstra, T. (2015). ‘Right Now, Sophie∗ Swims in the Pool?!’: Brain potentials of grammatical aspect processing. Frontiers in Psychology, 6, 1764. 26635673 10.3389/fpsyg.2015.01764 PMC4655232

[cogs13206-bib-0043] Foerde, K. , Knowlton, B. J. , & Poldrack, R. A. (2006). Modulation of competing memory systems by distraction. PNAS, 103(31), 11778–11783. 10.1073/pnas.0602659103 16868087 PMC1544246

[cogs13206-bib-0044] Foerde, K. , & Poldrack, R. A. (2009). Procedural learning in humans. In L. R. Squire (Ed.), Encyclopedia of neuroscience (pp. 1083–1091). Amsterdam: Academic Press.

[cogs13206-bib-0045] Francis, E. (2022). Gradient acceptability and linguistic theory. Oxford: Oxford University Press.

[cogs13206-bib-0046] Gabriel, A. , Maillart, C. , Stefaniak, N. , Lejeune, C. , Desmottes, L. , & Meulemans, T. (2013). Procedural learning in specific language impairment: Effects of sequence complexity. Journal of the International Neuropsychological Society, 19(3), 264–271. 10.1017/S1355617712001270 23298411

[cogs13206-bib-0047] Garcia, R. , Vouimba, R.‐M. , Baudry, M. , & Thompson, R. F. (1999). The amygdala modulates prefrontal cortex activity relative to conditioned fear. Nature, 402(6759), 294–296. 10580500 10.1038/46286

[cogs13206-bib-0048] Gonnerman, L. M. , Seidenberg, M. S. , & Andersen, E. S. (2007). Graded semantic and phonological similarity effects in priming: Evidence for a distributed connectionist approach to morphology. Journal of Experimental Psychology: General, 136(2), 323. 17500654 10.1037/0096-3445.136.2.323

[cogs13206-bib-0049] Gordon, P. C. , Hendrick, R. , & Levine, W. H. (2002). Memory‐load interference in syntactic processing. Psychological Science, 13(5), 425–430. 12219808 10.1111/1467-9280.00475

[cogs13206-bib-0050] Granena, G. (2013). Cognitive aptitudes for second language learning and the LLAMA language aptitude test. In G. Granena & M. H. Long (Eds.), Sensitive periods, language aptitude, and ultimate L2 attainment. Philadelphia: John Benjamins.

[cogs13206-bib-0051] Grosjean, F. O. (2010). Bilingual: Life and reality. Cambridge, MA: Harvard University Press.

[cogs13206-bib-1002] Hahn, U. , & Nakisa, R. C. (2000). German inflection: Single route or dual route? Cognitive Psychology, 41(4), 313–360. 11121259 10.1006/cogp.2000.0737

[cogs13206-bib-0052] Harm, M. W. , & Seidenberg, M. S. (2004). Computing the meanings of words in reading: Cooperative division of labor between visual and phonological processes. Psychological Review, 111(3), 662–720. 15250780 10.1037/0033-295X.111.3.662

[cogs13206-bib-0053] Henke, K. (2010). A model for memory systems based on processing modes rather than consciousness. Nature Reviews Neuroscience, 11(7), 523–532. doi: https://doi.org.ezproxye.bham.ac.uk/10.1038/nrn2850 20531422 10.1038/nrn2850

[cogs13206-bib-0054] Jackendoff, R. (2002). What's in the Lexicon? In S. Nooteboom , F. Weerman , & F. Wijnen (Eds.), Storage and computation in the language faculty (Vol. 30, pp. 23–58). Dordrecht: Kluwer.

[cogs13206-bib-0055] Javadi, A. H. , & Walsh, V. (2012). Transcranial direct current stimulation (tDCS) of the left dorsolateral prefrontal cortex modulates declarative memory. Brain Stimulation, 5(3), 231–241. 21840287 10.1016/j.brs.2011.06.007

[cogs13206-bib-0056] Jimenez, L. (Ed.) (2003). Attention and implicit learning (Vol. 48). Amsterdam: John Benjamins Publishing Company.

[cogs13206-bib-0057] Kidd, E. , & Kirjavainen, M. (2011). Investigating the contribution of procedural and declarative memory to the acquisition of past tense morphology: Evidence from Finnish. Language and Cognitive Processes, 26(4–6), 794–829. 10.1080/01690965.2010.493735

[cogs13206-bib-0058] Knowlton, B. J. , Siegel, A. L. M. , & Moody, T. D. (2017). Procedural learning in humans. In J. H. Byrne (Ed.), Learning and memory: A comprehensive reference ( 2nd ed., Vol. 3, pp. 295–312). Oxford: Academic Press.

[cogs13206-bib-0059] Knowlton, B. J. , & Squire, L. R. (1996). Artificial grammar learning depends on implicit acquisition of both abstract and exemplar‐specific information. Journal of Experimental Psychology: Learning, Memory, and Cognition, 22(1), 169. 8648284 10.1037//0278-7393.22.1.169

[cogs13206-bib-0060] Koenker, R. (2005). Quantile regression. Cambridge: Cambridge University Press.

[cogs13206-bib-0061] Langacker, R. W. (1987). Foundations of cognitive grammar (Vol. 1). Stanford: Stanford University Press.

[cogs13206-bib-0062] Llompart, M. , & Dabrowska, E. (2020). Explicit but not implicit memory predicts ultimate attainment in the native language. Frontiers in Psychology, 11. 10.3389/fpsyg.2020.569586 PMC7546274 33101138

[cogs13206-bib-0063] Lum, J. A. G. , Conti‐Ramsden, G. , Morgan, A. T. , & Ullman, M. T. (2014). Procedural learning deficits in specific language impairment (SLI): A meta‐analysis of serial reaction time task performance. Cortex, 51, 1–10. 24315731 10.1016/j.cortex.2013.10.011 PMC3989038

[cogs13206-bib-0064] Lum, J. A. G. , Conti‐Ramsden, G. , Page, D. , & Ullman, M. T. (2012). Working, declarative and procedural memory in specific language impairment. Cortex, 48, 1138–1154. 21774923 10.1016/j.cortex.2011.06.001 PMC3664921

[cogs13206-bib-0065] Luzzatti, C. , Mondini, S. , & Semenza, C. (2001). Lexical representation and processing of morphologically complex words: Evidence from the reading performance of an Italian agrammatic patient. Brain and language, 79(3), 345–359. 11781047 10.1006/brln.2001.2475

[cogs13206-bib-0066] MacDonald, K. B. (2008). Effortful control, explicit processing, and the regulation of human evolved predispositions. Psychological Review, 115(4), 1012–1031. 18954212 10.1037/a0013327

[cogs13206-bib-0067] Manns, J. R. , & Eichenbaum, H. (2006). Evolution of declarative memory. Hippocampus, 16(9), 795–808. 16881079 10.1002/hipo.20205

[cogs13206-bib-0068] Marantz, A. (2013). No escape from morphemes in morphological processing. Language and Cognitive Processes, 28(7), 905–916.

[cogs13206-bib-0069] Marcus, G. F. (1998). Rethinking eliminative connectionism. Cognitive Psychology, 37(3), 243–282. 9892549 10.1006/cogp.1998.0694

[cogs13206-bib-0070] Maslov, J. S. (1948). Vid i leksiçeskoe znaçenie glagola v russkom yazyke. Izvestiya Akademii Nauk SSSR. Otdelenie literatury i yazyka, 7(4), 303–316.

[cogs13206-bib-0071] Mathôt, S. , Schreij, D. , & Theeuwes, J. (2012). OpenSesame: An open‐source, graphical experiment builder for the social sciences. Behavior Research Methods, 44(2), 314–324. 10.3758/s13428-011-0168-7 22083660 PMC3356517

[cogs13206-bib-0072] Mayes, A. K. , & Reilly Morgan, A. T. (2015). Neural correlates of childhood language disorder: A systematic review. Developmental Medicine & Child Neurology, 57(8), 706–717. 10.1111/dmcn.12714 25692930

[cogs13206-bib-0073] McKee, R. D. , & Squire, L. R. (1993). On the development of declarative memory. Journal of Experimental Psychology: Learning, Memory, and Cognition, 19(2), 397–404. 8454964 10.1037//0278-7393.19.2.397

[cogs13206-bib-0074] Medimorec, S. , Mander, C. , & Risko, E. F. (2018). Memory demands in linguistic compensation. Quarterly Journal of Experimental Psychology, 71, 1234–1239. 10.1080/17470218.2017.1311353 28337947

[cogs13206-bib-0075] Medimorec, S. , Milin, P. , & Divjak, D. (2020). Frogs, apples, and sand: Effects of cognitive and demographic factors on letter fluency performance. Current Psychology, *41*, 2089.

[cogs13206-bib-0076] Medimorec, S. , Milin, P. , & Divjak, D. (2021). Working memory affects anticipatory behavior during implicit pattern learning. Psychological Research, *85*, 291. 10.1007/s00426-019-01251-w 31562540

[cogs13206-bib-0077] Milin, P. , Divjak, D. , & Baayen, R. H. (2017). A learning perspective on individual differences in skilled reading: Exploring and exploiting orthographic and semantic discrimination cues. Journal of Experimental Psychology: Learning, Memory, and Cognition, 43(11), 1730–1751. 10.1037/xlm0000410 28383952

[cogs13206-bib-0079] Mishkin, M. , Malamut, B. , & Bachevalier, J. (1984). Memories and habits: Two neural systems. In G. Lynch , J. L. McGaugh , & N. M. Weinberger (Eds.), Neurobiology of learning and memory (pp. 65–77). New York: Guilford.

[cogs13206-bib-0080] Moody, T. D. , Bookheimer, S. Y. , Vanek, Z. , & Knowlton, B. J. (2004). An implicit learning task activates medial temporal lobe in patients with Parkinson's disease. Behavioral Neuroscience, 118(2), 438. 15113271 10.1037/0735-7044.118.2.438

[cogs13206-bib-0081] Mulligan, N. W. (1997). Attention and implicit memory tests: The effects of varying attentional load on conceptual priming. Memory & Cognition, 25(1), 11–17. 10.3758/BF03197281 9046866

[cogs13206-bib-0082] Newman, A. J. , Supalla, T. , Hauser, P. , Newport, E. L. , & Bavelier, D. (2010). Dissociating neural subsystems for grammar by contrasting word order and inflection. Proceedings of the National Academy of Sciences, 107(16), 7539–7544. 10.1073/pnas.1003174107 PMC2867749 20368422

[cogs13206-bib-0083] Opitz, B. , & Friederici, A. D. (2003). Interactions of the hippocampal system and the prefrontal cortex in learning language‐like rules. NeuroImage, 19, 1730–1737. 12948727 10.1016/s1053-8119(03)00170-8

[cogs13206-bib-0084] Pinker, S. (1984). Language learnability and language acquisition. Cambridge, MA: Harvard University Press.

[cogs13206-bib-0085] Pinker, S. (1991). Rules of language. Science, 253(5019), 530–535. 1857983 10.1126/science.1857983

[cogs13206-bib-0086] Pinker, S. (1999). Words and rules: The ingredients of language. New York: Basic Books.

[cogs13206-bib-0087] Plaut, D. , McClelland, J. , Seidenberg, M. , & Patterson, K. (1996). Visual word recognition: Are two routes really necessary. Psychological Review, 103, 56–115. 8650300 10.1037/0033-295x.103.1.56

[cogs13206-bib-0088] Plaut, D. C. , & Gonnerman, L. M. (2000). Are non‐semantic morphological effects incompatible with a distributed connectionist approach to lexical processing? Language and Cognitive Processes, 154, 445–485.

[cogs13206-bib-0089] Poldrack, R. A. , Clark, J. , Pare‐Blagoev, E. J. , Shohamy, D. , Creso Moyano, J. , Myers, C. , & Gluck, M. A. (2001). Interactive memory systems in the human brain. Nature, 414(6863), 546–550. 10.1038/35107080 11734855

[cogs13206-bib-0090] Poldrack, R. A. , Sabb, F. W. , Foerde, K. , Tom, S. M. , Asarnow, R. F. , Bookheimer, S. Y. , & Knowlton, B. J. (2005). The neural correlates of motor skill automaticity. Journal of Neuroscience, 25(22), 5356–5364. 10.1523/JNEUROSCI.3880-04.2005 15930384 PMC6725010

[cogs13206-bib-0091] Prull, M. W. , Lawless, C. , Marshall, H. M. , & Sherman, A. T. (2016). Effects of divided attention at retrieval on conceptual implicit memory. Frontiers in Psychology, 7(5). doi: 10.3389/fpsyg.2016.00005 PMC4720745 26834678

[cogs13206-bib-0092] R Core Team . (2020). R: A language and environment for statistical computing (Version 3.6.2). Vienna, Austria: R Foundation for Statistical Computing. Retrieved from https://www.R‐project.org/

[cogs13206-bib-0093] Reber, A. S. (1989). Implicit learning and tacit knowledge. Journal of Experimental Psychology: General, 118, 219–235.

[cogs13206-bib-0094] Reber, P. J. (2008). Cognitive neuroscience of declarative and nondeclarative memory. Advances in Psychology, 139, 113–123.

[cogs13206-bib-0095] Reber, P. J. , Knowlton, B. J. , & Squire, L. R. (1996). Dissociable properties of memory systems: Differences in the flexibility of declarative and nondeclarative knowledge. Behavioral Neuroscience, 110(5), 861. 8918990 10.1037//0735-7044.110.5.861

[cogs13206-bib-0096] Richmond, J. , & Nelson, C. A. (2007). Accounting for change in declarative memory: A cognitive neuroscience perspective. Developmental Review, 27(3), 349–373. doi: 10.1016/j.dr.2007.04.002 18769510 PMC2094108

[cogs13206-bib-0097] Rogers, V. , Meara, P. , Barnett‐Legh, T. , Curry, C. , & Davie, E. (2017). Examining the LLAMA aptitude tests. Journal of the European Second Language Association, 1(1), 49–60. doi: 10.22599/jesla.24

[cogs13206-bib-0098] Rudas, T. (2018). Lectures on categorical data analysis. New York: Springer.

[cogs13206-bib-0099] Rugg, M. D. , & Yonelinas, A. P. (2003). Human recognition memory: A cognitive neuroscience perspective. Trends in Cognitive Sciences, 7(7), 313–319. 12860190 10.1016/s1364-6613(03)00131-1

[cogs13206-bib-0100] Rumelhart, D. E. , & McClelland, J. (1986). On learning the past tense of English verbs. In D. E. Rumelhart & J . McClelland (Eds.), Parallel distributed processing (Vol. 2, pp. 216–271). Cambridge, MA: MIT Press.

[cogs13206-bib-0101] Ryle, G. (1949) [2002]. The concept of mind. Chicago: University of Chicago Press.

[cogs13206-bib-0102] Schacter, D. L. (1987). Implicit memory: History and current status. Journal of Experimental Psychology: Learning, Memory, and Cognition, 13, 501–518. 10.1037//0278-7393.11.3.501 3160813

[cogs13206-bib-0103] Schmidtke, D. , Matsuki, K. , & Kuperman, V. (2017). Surviving blind decomposition: A distributional analysis of the time‐course of complex word recognition. Journal of Experimental Psychology: Learning, Memory, and Cognition, 43(11), 1793. 28447810 10.1037/xlm0000411 PMC5659973

[cogs13206-bib-0104] Segalowitz, S. , & Segalowitz, N. (1993). Skilled performance, practice, and the differentiation of speed‐up from automatization effects: Evidence from second language word recognition. Applied Psycholinguistics, 14(3), 369–385. 10.1017/S0142716400010845

[cogs13206-bib-0105] Segalowitz, S. , Segalowitz, N. , & Wood, A. (1998). Assessing the development of automaticity in second language word recognition. Applied Psycholinguistics, 19(1), 53–67. 10.1017/S0142716400010572

[cogs13206-bib-0106] Seidenberg, M. S. , & Gonnerman, L. M. (2000). Explaining derivational morphology as the convergence of codes. Trends in Cognitive Sciences, 4(9), 353–361. 10962617 10.1016/s1364-6613(00)01515-1

[cogs13206-bib-0107] Siegelman, M. , Blank, I. A. , Mineroff, Z. , & Fedorenko, E. (2019). An attempt to conceptually replicate the dissociation between syntax and semantics during sentence comprehension. Neuroscience, 413, 219–229. 10.1016/j.neuroscience.2019.06.003 PMC6661197 31200104

[cogs13206-bib-0108] Siegelman, N. , & Frost, R. (2015). Statistical learning as an individual ability: Theoretical perspectives and empirical evidence. Journal of Memory and Language, 81, 105–120. 10.1016/j.jml.2015.02.001 25821343 PMC4371530

[cogs13206-bib-0109] Spataro, P. , Cestari, V. , & Rossi‐Arnaud, C. (2011). The relationship between divided attention and implicit memory: A meta‐analysis. Acta Psychologica, 136, 329–339. doi: 10.1016/j.actpsy.2010.12.007 21257140

[cogs13206-bib-0110] Squire, L. R. (1992). Declarative and nondeclarative memory: Multiple brain systems supporting learning and memory. Journal of Cognitive Neuroscience, 4(3), 232–243. 23964880 10.1162/jocn.1992.4.3.232

[cogs13206-bib-0111] Squire, L. R. (2004). Memory systems of the brain: A brief history and current perspective. Neurobiology of Learning and Memory, 82(3), 171–177. 15464402 10.1016/j.nlm.2004.06.005

[cogs13206-bib-0112] Squire, L. R. , Bayley, P. J. , & Smith, C. N. (2009). Amnesia: Declarative and nondeclarative memory. In L. R. Squire (Ed.), Encyclopedia of neuroscience (pp. 289–294). Oxford: Academic Press.

[cogs13206-bib-0113] Squire, L. R. , & Kandel, E. R. (2009). Memory : From mind to molecules. Totnes: Roberts and Company.

[cogs13206-bib-0114] Squire, L. R. , Knowlton, B. J. , & Musen, G. (1993). The structure and organization of memory. Annual Review of Psychology, 44, 453–495. 10.1146/annurev.ps.44.020193.002321 8434894

[cogs13206-bib-0115] Squire, L. R. , & Wixted, J. T. (2011). The cognitive neuroscience of human memory since H.M. Annual Review of Neuroscience, 34, 259–288. 10.1146/annurev-neuro-061010-113720 PMC3192650 21456960

[cogs13206-bib-0116] Squire, L. R. , & Zola‐Morgan, S. (1991). The medial temporal lobe memory system. Science, 253(5026), 1380–1386. 1896849 10.1126/science.1896849

[cogs13206-bib-0117] Sun, R. , Slusarz, P. , & Terry, C. (2005). The interaction of the explicit and the implicit in skill learning: A dual‐process approach. Psychological Review, 112(1), 159–192. 15631592 10.1037/0033-295X.112.1.159

[cogs13206-bib-0118] Thompson, R. F. , & Krupa, D. J. (1994). Organization of memory traces in the mammalian brain. Annual Review of Neuroscience, 17(1), 519–549. 10.1146/annurev.ne.17.030194.002511 8210186

[cogs13206-bib-0119] Tomaschek, F. , Tucker, B. V. , Fasiolo, M. , & Baayen, R. H. J. L. V. (2018). Practice makes perfect: The consequences of lexical proficiency for articulation. 4(s2).

[cogs13206-bib-0120] Tomasello, M. (1992). First verbs: A case study of early grammatical development. Cambridge: Cambridge University Press.

[cogs13206-bib-0121] Ullman, M. T. (2004). Contributions of memory circuits to language: The declarative/procedural model. Cognition, 92(1), 231–270. 15037131 10.1016/j.cognition.2003.10.008

[cogs13206-bib-0122] Ullman, M. T. (2016). The declarative/procedural model: A neurobiological model of language learning, knowledge, and use. In G. Hickok & S. L. Small (Eds.), Neurobiology of language (pp. 953–968). Academic Press. https://www.sciencedirect.com/science/article/pii/B9780124077942000766?via%3Dihub

[cogs13206-bib-0123] Ullman, M. T. , Earle, F. S. , Walenski, M. , & Janacsek, K. (2020). The neurocognition of developmental disorders of language. Annual Review of Psychology, 71, 389–417. 10.1146/annurev-psych-122216-011555 31337273

[cogs13206-bib-0124] Ullman, M. T. , & Lovelett, J. T. (2018). Implications of the declarative/procedural model for improving second language learning: The role of memory enhancement techniques. *Second Language Research*, 34(1), 39–65. 10.1177/0267658316675195

[cogs13206-bib-0125] Vakil, E. , Bloch, A. , & Cohen, H. (2017). Anticipation measures of sequence learning: Manual versus oculomotor versions of the serial reaction time task. Quarterly Journal of Experimental Psychology, 70(3), 579–589. 10.1080/17470218.2016.1172095 27042771

[cogs13206-bib-0126] Van Rij, J. , Wieling, M. , Baayen, R. H. , & van Rijn, H. (2020). itsadug: Interpreting time series and autocorrelated data using GAMMs (Version 2.4): R statistical group.

[cogs13206-bib-0127] Varkanitsa, M. , & Caplan, D. (2018). On the association between memory capacity and sentence comprehension: Insights from a systematic review and meta‐analysis of the aphasia literature. Journal of Neurolinguistics, 28, 4–25. doi: 10.1016/j.jneuroling.2018.03.003

[cogs13206-bib-0128] Vendler, Z. (1957). Verbs and times. The Philosophical Review, 66(6), 143–160.

[cogs13206-bib-0129] Wagner, A. D. , Koutstaal, W. , Maril, A. , Schacter, D. L. , & Buckner, R. L. (2000). Task‐specific repetition priming in left inferior prefrontal cortex. Cerebral Cortex, 10(12), 1176–1184. 11073867 10.1093/cercor/10.12.1176

[cogs13206-bib-0130] Wagner, A. D. , Schacter, D. L. , Rotte, M. , Koutstaal, W. , Maril, A. , Dale, A. M. , … Buckner, R. L. (1998). Building memories: Remembering and forgetting of verbal experiences as predicted by brain activity. Science, 281(5380), 1188–1191. 9712582 10.1126/science.281.5380.1188

[cogs13206-bib-0131] Walenski, M. , Europa, E. , Caplan, D. , & Thompson, C. K. (2019). Neural networks for sentence comprehension and production: An ALE‐based meta‐analysis of neuroimaging studies. Human Brain Mapping, 40(8), 2275–2304. 10.1002/hbm.24523 30689268 PMC6597252

[cogs13206-bib-0132] Waters, G. , Caplan, D. , & Yampolsky, S. (2003). On‐line syntactic processing under concurrent memory load. Psychonomic Bulletin and Review: A Journal of the Psychonomic Society, Inc, 10(1), 88–95. 10.3758/BF03196471 12747494

[cogs13206-bib-0133] West, G. , Vadillo, M. A. , Shanks, D. R. , & Hulme, C. (2018). The procedural learning deficit hypothesis of language learning disorders: We see some problems. Developmental Science, 21(2), e12552. 10.1111/desc.12552 28256101 PMC5888158

[cogs13206-bib-0134] Wilkinson, L. , & Shanks, D. R. (2004). Intentional control and implicit sequence learning. Journal of Experimental Psychology: Learning, Memory & Cognition, 30, 354–369. 14979810 10.1037/0278-7393.30.2.354

[cogs13206-bib-0135] Willingham, D. B. , Nissen, M. J. , & Bullemer, P. (1989). On the development of procedural knowledge. Journal of Experimental Psychology: Language, Memory, Cognition, 15(6), 1047–1060. 10.1037//0278-7393.15.6.1047 2530305

[cogs13206-bib-0136] Wolters, G. , & Prinsen, A. (1997). Full versus divided attention and implicit memory performance. Memory & Cognition, 25(6), 764–771. 10.3758/BF03211319 9421561

[cogs13206-bib-0137] Wood, S. N. (2006). Generalized additive models. Boca Raton, FL: Chapman & Hall.

[cogs13206-bib-0138] Wood, S. N. (2011). Fast stable restricted maximum likelihood and marginal likelihood estimation of semiparametric generalized linear models. Journal of the Royal Statistical Society: Series B (Statistical Methodology), 73(1), 3–36.

[cogs13206-bib-0139] Woodruff‐Pak, D. S. , & Lemieux, S. K. (2001). The cerebellum and associative learning: Parallels and contrasts in rabbits and humans. In J. E. Steinmetz , M. A. Gluck , & P. R. Solomon (Eds.), Model systems and the neurobiology of associative learning: A Festschrift in Honor of Richard F. Thompson (pp. 271–294). Mahwah: Erlbaum.

