# Supplementary material for: Behavioral Signatures of Memory Resources for Language: Looking beyond the Lexicon/Grammar Divide
Source: Cogn Sci. 2022 Nov 10;46(11):e13206. doi: 10.1111/cogs.13206 (PMC9787600; doi:10.1111/cogs.13206)
Supplement: Supplementary file 1 — Supplementary information [file COGS-46-e13206-s002.docx]

**SupMat 1** **Detailed presentation of stimulus types**

*Note: in the examples given below, the gloss contains the information about the error, but the translation is correct because there is no straightforward equivalent to these errors in English.*

Polish has 7 grammatical **cases**, expressed by inflectional endings of nouns, pronouns, adjectives, adjectival participles and numerals. The main function of cases is to indicate the relations between the elements of the sentence. For instance, nominative is primarily used to indicate the subject of the sentence, while genitive indicates possession, similarly to the English ''s' or 'of'. The choice of a case ending depends on the gender and number, which inflates the number of case endings. This, in combination with syncretism of some endings and many exceptions, makes the system difficult to acquire for second language learners. We included an incorrect sentence for 6 cases (the vocative case was excluded because of its limited usage), where a noun was used in the incorrect case, for example (1), where the instrumental *motywacją* is used instead of the accusative *motywacji*:

(1) *Zwykle po pracy brakuje mi motywacją żeby iść na siłownię.*

usually after work;loc lack;3sg;prs I;gen motivation;inst to go on gym;acc

‘Usually after work I lack the motivation to go to the gym’

Erroneous **subordination** was exemplified through three *że*-introduced clauses in which a wrong form of the subordinate verb was used, as in (2) where the infinitive is used instead of the past tense, as well as three *żeby*-introduced clauses, as in (3), with the same type of error.

(2) *Mężczyzna zaprzeczył, że to on popełnić. morderstwo.*

man;nom denied;3sg;m;pst that it he;nom commit murder;acc

‘The man denied that it was him who committed the murder’

(3) *Staramy się, żeby nasi widzowie mieć dostęp do najnowszych filmów.*

try;1pl;prs refl that our viewer;pl have access to latest film;pl

We’re trying to make sure our viewers have access to the latest films

**Collocation** errors, where the word choice was incorrect, were represented by three sentences where an incorrect verb was used and three sentences where an incorrect noun was used. Take for example sentence (4) where *zapis drogowy* is used instead of *przepis drogowy*.

(4) *Przyczyna wypadków to nieprzestrzeganie zapisów drogowych.*

cause;nom accident;gen it ignoring writing;pl traffic;adj

‘Ignoring the road traffic regulations was the cause of the accident’

**Aspect** errors. All verbs in Polish (with the exception of a few biaspectual verbs) are said to belong to one of the two aspectual categories: perfective or imperfective. Perfective verbs are usually described as expressing completion of an action, as opposed to an 'ongoing' interpretation of the imperfective aspect. The choice of aspect is limited in certain contexts. For instance, perfective cannot be used in present tense or with certain temporal expressions, such as 'przez trzy godziny' (for three hours. Similarly, imperfective verb is incompatible with adverbs such as 'wkrótce' (soon'). The erroneous sentences of this type, in which perfective was used instead of imperfective and vice versa, looked like the example in (5), where the imperfective *pić* is used instead of perfective *wypić*.

(5) *Lubię od czasu do czasu pić piwo czy dwa.*

like;1sg;prs from time;gen to time;gen drink beer or two

‘I like to have a beer or two from time to time’

Half of all filler items contained one of four different types of errors (again, six sentences per type). We included preposition errors, where the sentence contained an incorrect preposition as in (6), number errors, where the number marked on the verb that did not match the subject in number as in (7), gender errors, where three sentences contained verbs whose gender did not match the subject as in (8), and three sentences that contained adjectives whose gender did not match the noun as in (9), and, finally, conjunction errors, where sentences contained an incorrect conjunction as in (10).

(6) *W tej restauracji można płacić kartą po bankomatu.*

in this restaurant;loc can pay card;inst after cash.mashine;gen

‘This restaurant accepts card payments’

(7) *Kasia i Tomek pracuje w firmie informatycznej dwa lata.*

Kasia and Tomek work;3sg in company;loc IT;adj two year;pl

‘Kasia and Tomek have been working for an IT company for two years.’

(8) *Wczorajsze spotkanie zarządu trwała bardzo długo.*

yesterday;adj meeting board;gen last;3sg;f very long

‘Yesterday’s board meeting lasted very long.’

(9) *Pogoda w ten weekend zapowiada się fantastyczny.*

weather in this weekend bode;3sg;f refl fantastic

‘The weather forecast for this weekend is fantastic’

(10) *Do banku jest niedaleko, albo zdążę przed wykładem.*

to bank;acc is close or make.it;1sg;prs before lecture;inst

‘The bank is very close, I can make it there before the lecture.’
