# Supplementary material for: Behavioral Signatures of Memory Resources for Language: Looking beyond the Lexicon/Grammar Divide
Source: Cogn Sci. 2022 Nov 10;46(11):e13206. doi: 10.1111/cogs.13206 (PMC9787600; doi:10.1111/cogs.13206)
Supplement: Supplementary file 2 — Table A. Generalized Additive Mixed Model fitted to the grammaticality judgment decision latencies (log‐transformed). Table B. Bayesian Generalized Additive Mixed Model fitted to the grammaticality judgment decision latencies (log‐transformed), using 4 chains with 4000 iterations each. Table C. Additive Quantile Mixed Model fitted to the rolling standard deviations over the grammaticality judgment decision times. Table D. Bayesian Additive Quantile Mixed Model fitted to the rolling standard deviations over the grammaticality judgment decision times, using 4 chains with 4000 iterations each. [file COGS-46-e13206-s003.docx]

**Table A.** Generalized Additive Mixed Model fitted to the grammaticality judgment decision latencies (log-transformed).

**gam**(Rt.log ~

(Type + Llama_F + SRT) * Condition +

TrialOrder.z +

s(Item, bs='re') +

s(TrialOrder.z, Participant, bs='fs', m=1),

...)

**Parametric coefficients:**

|  | **Estimate** | **Std. Error** | **t value** | **Pr(>\|t\|)** |
| --- | --- | --- | --- | --- |
| (Intercept) | 6.237 | 0.129 | 48.371 | < 2e-16 |
| Type(case) | 0.082 | 0.093 | 0.874 | 0.382 |
| Type(collocation) | 0.146 | 0.094 | 1.561 | 0.119 |
| Type(aspect) | 0.285 | 0.094 | 3.039 | 0.002 |
| Llama_F(Avg.High) | -0.339 | 0.126 | -2.683 | 0.007 |
| Llama_F(High) | -0.275 | 0.111 | -2.484 | 0.013 |
| SRT(Avg.Slow) | 0.015 | 0.126 | 0.119 | 0.905 |
| SRT(Avg.Fast) | 0.024 | 0.130 | 0.183 | 0.855 |
| SRT(Fast) | 0.057 | 0.132 | 0.430 | 0.667 |
| Condition(CT) | 0.294 | 0.058 | 5.050 | < 0.0001 |
| TrialOrder.z | -0.031 | 0.016 | -2.001 | 0.046 |
| Type(case):Condition(CT) | -0.039 | 0.054 | -0.725 | 0.468 |
| Type(collocation):Condition(CT) | 0.041 | 0.055 | 0.748 | 0.455 |
| Type(aspect):Condition(CT) | -0.116 | 0.055 | -2.111 | 0.035 |
| Llama_F(Avg.High):Condition(CT) | 0.156 | 0.059 | 2.653 | 0.008 |
| Llama_F(High):Condition(CT) | 0.153 | 0.048 | 3.195 | 0.001 |
| SRT(Avg.Slow):Condition(CT) | 0.098 | 0.055 | 1.768 | 0.077 |
| SRT(Avg.Fast):Condition(CT) | 0.121 | 0.056 | 2.173 | 0.030 |
| SRT(Fast):Condition(CT) | -0.154 | 0.058 | -2.670 | 0.008 |

**Approximate significance of smooth terms:**

|  | **edf** | **Ref.df** | **F** | **p-value** |
| --- | --- | --- | --- | --- |
| s(Item) | 83.16 | 92 | 7.824 | <2e-16 |
| s(TrialOrder.z,Participant) | 93.45 | 399 | 2.213 | <2e-16 |

R-sq.(adj) = 0.363; Deviance explained = 39.7%;

GCV = 0.35723; Scale est. = 0.33814; n = 3660

**Table B.** Bayesian Generalized Additive Mixed Model fitted to the grammaticality judgment decision latencies (log-transformed), using 4 chains with 4000 iterations each.

**brm**(RT.log ~

(Type + Llama_F + SRT) * Condition +

TrialOrder.z +

s(Item, bs='re') +

s(TrialOrder.z, Participant, bs='fs', m=1),

chains=4, iter=4000, cores=4, control=list(adapt_delta=.95),

...)

**Smooth Terms:**

|  | **Estimate** | **Est.Error** | **l-95% CI** | **u-95% CI** |
| --- | --- | --- | --- | --- |
| sds(Item) | 0.28 | 0.02 | 0.24 | 0.33 |
| sds(TrialOrder.z,Participant)1 | 0.31 | 0.05 | 0.22 | 0.41 |
| sds(TrialOrder.z,Participant)2 | 2.07 | 0.26 | 1.62 | 2.63 |

**Population-Level Effects:**

|  | **Estimate** | **Est.Error** | **l-95% CI** | **u-95% CI** |
| --- | --- | --- | --- | --- |
| (Intercept) | 6.23 | 0.13 | 5.97 | 6.49 |
| Type(case) | 0.09 | 0.09 | -0.08 | 0.26 |
| Type(collocation) | 0.15 | 0.09 | -0.03 | 0.33 |
| Type(aspect) | 0.29 | 0.09 | 0.11 | 0.47 |
| Llama_F(Avg.High) | -0.34 | 0.13 | -0.59 | -0.09 |
| Llama_F(High) | -0.27 | 0.11 | -0.49 | -0.05 |
| SRT(Avg.Slow) | 0.02 | 0.13 | -0.23 | 0.27 |
| SRT(Avg.Fast) | 0.03 | 0.13 | -0.23 | 0.28 |
| SRT(Fast) | 0.06 | 0.13 | -0.2 | 0.32 |
| Condition(CT) | 0.29 | 0.06 | 0.18 | 0.41 |
| TrialOrder.z | -0.03 | 0.02 | -0.06 | 0.00 |
| Type(case):Condition(CT) | -0.04 | 0.05 | -0.14 | 0.07 |
| Type(collocation):Condition(CT) | 0.04 | 0.06 | -0.07 | 0.15 |
| Type(aspect):Condition(CT) | -0.12 | 0.05 | -0.22 | -0.01 |
| Llama_F(Avg.High):Condition(CT) | 0.16 | 0.06 | 0.04 | 0.27 |
| Llama_F(High):Condition(CT) | 0.15 | 0.05 | 0.06 | 0.25 |
| SRT(Avg.Slow):Condition(CT) | 0.1 | 0.05 | -0.01 | 0.2 |
| SRT(Avg.Fast):Condition(CT) | 0.12 | 0.06 | 0.01 | 0.23 |
| SRT(Fast):Condition(CT) | -0.15 | 0.06 | -0.26 | -0.04 |

**Family Specific Parameters:**

|  | **Estimate** | **Est.Error** | **l-95% CI** | **u-95% CI** |
| --- | --- | --- | --- | --- |
| sigma | 0.58 | 0.01 | 0.57 | 0.6 |

**Table C.** Additive Quantile Mixed Model fitted to the rolling standard deviations over the grammaticality judgment decision times.

**mqgam**(rollSD ~

Type + Condition +

TrialOrder.z +

s(Item, bs='re') +

s(TrialOrder.z, Participant, bs='fs', m=1),

qu=0.5, argGam=list(method='ML'),

...)

**Parametric coefficients:**

|  | **Estimate** | **Std. Error** | **z value** | **Pr(>\|z\|)** |
| --- | --- | --- | --- | --- |
| (Intercept) | 0.450 | 0.021 | 21.788 | < 2e-16 |
| Type(case) | 0.050 | 0.017 | 2.958 | 0.003 |
| Type(aspect) | 0.117 | 0.017 | 6.752 | < 0.0001 |
| Type(collocation) | 0.150 | 0.017 | 8.576 | < 2e-16 |
| Condition(CT) | -0.064 | 0.011 | -6.105 | < 0.0001 |
| TrialOrder.z | 0.009 | 0.007 | 1.202 | 0.229 |

**Approximate significance of smooth terms:**

|  | **edf** | **Ref.df** | **Chi.sq** | **p-value** |
| --- | --- | --- | --- | --- |
| s(Item) | 25.380 | 92.000 | 37.290 | 0.002 |
| s(TrialOrder.z,Participant) | 79.840 | 404.000 | 469.790 | < 2e-16 |

R-sq.(adj) = 0.141; Deviance explained = 15.4%;

-ML = 997.1; Scale est. = 1; n = 3519

**Table D.** Bayesian Additive Quantile Mixed Model fitted to the rolling standard deviations over the grammaticality judgment decision times, using 4 chains with 4000 iterations each.

**brm**(**bf**(rollSD ~

Type + Condition +

TrialOrder.z +

s(Item, bs='re') +

s(TrialOrder.z, Participant, bs='fs', m=1),

quantile=0.5), family=asym_laplace(),

chains=4, iter=4000, cores=4,

...)

**Smooth Terms:**

|  | **Estimate** | **Est.Error** | **l-95% CI** | **u-95% CI** |
| --- | --- | --- | --- | --- |
| sds(Item) | 0.04 | 0.01 | 0.02 | 0.05 |
| sds(TrialOrder.z,Participant)1 | 0.31 | 0.05 | 0.21 | 0.42 |
| sds(TrialOrder.z,Participant)2 | 1.52 | 0.18 | 1.2 | 1.93 |

**Population-Level Effects:**

|  | **Estimate** | **Est.Error** | **l-95% CI** | **u-95% CI** |
| --- | --- | --- | --- | --- |
| (Intercept) | 0.44 | 0.02 | 0.39 | 0.48 |
| Type(case) | 0.05 | 0.02 | 0.02 | 0.09 |
| Type(aspect) | 0.12 | 0.02 | 0.09 | 0.16 |
| Type(collocation) | 0.16 | 0.02 | 0.12 | 0.2 |
| Condition(CT) | -0.06 | 0.01 | -0.08 | -0.04 |
| TrialOrder.z | 0.01 | 0.01 | -0.01 | 0.03 |

**Family Specific Parameters:**

|  | **Estimate** | **Est.Error** | **l-95% CI** | **u-95% CI** |
| --- | --- | --- | --- | --- |
| sigma | 0.12 | 0 | 0.12 | 0.12 |
| quantile | 0.5 | 0 | 0.5 | 0.5 |
