# Supplementary material for: Behavioral Signatures of Memory Resources for Language: Looking beyond the Lexicon/Grammar Divide
Source: Cogn Sci. 2022 Nov 10;46(11):e13206. doi: 10.1111/cogs.13206 (PMC9787600; doi:10.1111/cogs.13206)
Supplement: Supplementary file 3 — Supplementary information [file COGS-46-e13206-s001.docx]

**SET 1**

| **Sentence** | **Error type** | **Correct** | **Translation** |
| --- | --- | --- | --- |
| To będzie najlepsza polska restauracja w Warszawie. | case | 1 | This will be the best Polish restaurant in Warsaw. |
| Michał to mój największy wrogiem z podstawówki. | case | 0 | Michał is my worst enemy from primary school. |
| Wczoraj wieczorem zaginął pies mojej sąsiadki. | case | 1 | Yesterday evening my neighbour’s dog got lost. |
| Następnym razem powinieneś dodać mniej pieprzem. | case | 0 | Next time you should add less pepper. |
| Na rocznicę chciałbym dać rodzicom coś wyjątkowego. | case | 1 | For their anniversary, I would like to give my parents something special. |
| Jej babcia robi swoim ulubionym wnukami swetry na drutach. | case | 0 | Her grandmother knits jumpers for her grandchildren. |
| Wieczorami, po powrocie z biura, pisał opowiadania. | case | 1 | In the evenings, having returned from the office, he wrote short stories. |
| Poznałam wczoraj naprawdę wyjątkową osobie. | case | 0 | Yesterday, I met a really special person |
| Jeszcze nigdy nie jechałam takim szybkim pociągiem. | case | 1 | I have never been on such a fast train. |
| Robotnicy przez dwie godziny tłukli młotków w ścianę. | case | 0 | The workers were pounding the wall with hammers for two hours. |
| Na zjeździe omawiano najważniejsze problemy okręgu. | case | 1 | During the congress, the most important problems of the region were discussed. |
| Pracownicy firmy ustawili maszynę przy ścianą domu. | case | 0 | The workers from the company put the machine next to the wall of the house |
| Muszę umówić się na wizytę u dentysty. | set_phrase | 1 | I have to set up an appointment with a dentist. |
| Cztery lata temu Paweł wyjechał do Niemiec żeby robić karierę. | set_phrase | 1 | For years ago, Paweł went to Germany to start a career. |
| Michał Kowalski pełni funkcję dyrektora firmy już drugą dekadę. | set_phrase | 1 | Michał Kowalski has been working as a director of the company for two decades. |
| W forumularzu proszę podać swoje nazwisko i datę urodzenia. | set_phrase | 1 | In the form, please provide your name and date of birth. |
| Po pracy zwykle idę na szybkie zakupy do galerii handlowej. | set_phrase | 1 | After work, I usually do quick shopping in the shopping centre. |
| Tomek interesuje się medycyną naturalną i akupunkturą. | set_phrase | 1 | Tomek is interested in natural medicine and acupuncture. |
| Weź ze sobą kurtkę bo chucha silny wiatr i pada deszcz. | set_phrase | 0 | Take a jacket with you, it’s raining and the wind is blowing. |
| To proste ćwiczenie pomoże ci szybko wypalić dużo kalorii. | set_phrase | 0 | This simple exercise will help you quickly burn a lot of calories. |
| Mama kazała mi ubrać ziemniaki i marchew na obiad. | set_phrase | 0 | Mum told me to peel the potatoes and carrots for dinner. |
| Najbliższa puszka pocztowa znajduje się obok sklepu w centrum miasta. | set_phrase | 0 | The nearest post box is next to the shop in the city centre. |
| Do zestawu komputerowego zostały dołączone nauszniki bezprzewodowe. | set_phrase | 0 | Wireless headphones are included in this computer set. |
| W przyszłym tygodniu idę do optyka po nowe okulary kontaktowe. | set_phrase | 0 | Next week, I’m going to the optician for new corrective lenses. |
| Turyści skarżyli się, że las wygląda na zniszczony. | aspect_tense | 1 | The tourists complained that the forest looks destroyed. |
| Janek pięć lat mieszkał w wiosce pod Kielcami. | aspect_tense | 1 | Janek lived in a village next to Kielce for five years. |
| Aby móc to zrobić, potrzebne jest im więcej pieniędzy. | aspect_tense | 1 | To be able to do it, they need more money. |
| W tej sytuacji, im więcej drzew się wytnie, tym lepiej. | aspect_tense | 1 | In this situation, the more trees they’ll cut down, the better. |
| Opowiem mu twoją historię jak tylko go zobaczę. | aspect_tense | 1 | I’ll them your story as soon as I see him. |
| Wczoraj nauczyłem się pięćdziesięciu nowych hiszpańskich słów. | aspect_tense | 1 | Yesterday, I learnt 50 new Spanish words. |
| Od kilku dni, w internecie krąży film, który zdobywał ogromną popularność. | aspect_tense | 0 | Since a few days ago, a video has been circulated on the internet. |
| Dziennikarz przeprowadzał i upowszechnił znany wielu prosty eksperyment. | aspect_tense | 0 | The journalist conducted and published a simple experiment, known to many. |
| Według szacunków, równość płci na rynku pracy będziemy osiągać dopiero za 170 lat. | aspect_tense | 0 | According to the estimations, the equality of genders will be reached in 170 years. |
| W trakcie wakacji całymi dniami usiadł przed komputerem. | aspect_tense | 0 | During holidays, he was spending all days in front of the computer. |
| Musisz jak najszybciej zmieniać tryb życia. | aspect_tense | 0 | You have to change your lifestyle as soon as possible. |
| Mimo wysiłków, lekarz leczył ją tylko na jakiś czas. | aspect_tense | 0 | Despite the efforts, the doctor cured her only temporarily. |
| Nie mogę uwierzyć, że udało im się zebrać tyle pieniędzy. | syntax | 1 | I can’t believe they’ve managed to raise so much money. |
| Wydaje mi się, że ten mężczyzna jest naszym nauczycielem. | syntax | 1 | I think this man is our teacher. |
| Po ogłoszeniu wyników było wiadomo, że wszyscy zdali. | syntax | 1 | After the results have been published, it turned out that everyone passed. |
| Czekałem na ciebie, żeby opowiedzieć ci o tym co się stało. | syntax | 1 | I was waiting for you to tell you what happened. |
| Niestety nie mieliśmy już czasu, żeby zwiedzić Warszawę. | syntax | 1 | Unfortunately, there was no time left to visit Warsaw. |
| Wstałem wcześnie rano i poszedłem do piekarni, żeby kupić chleb i bułki. | syntax | 1 | I woke up early and went to the bakery to buy bread and rolls. |
| Korek spowodował, że autobusy zacząć się spóźniać. | syntax | 0 | The traffic jam caused bus delays. |
| W wywiadzie powiedziała, że napisać książkę w dwa miesiące. | syntax | 0 | In the interview, she said that she wrote the book in two months. |
| Stracił wszystko przez to, że grać w pokera. | syntax | 0 | He lost everything because he played poker. |
| Wyjechał w góry, żeby odciął się od wszystkiego. | syntax | 0 | He went to the mountains to cut himself off everything. |
| Rodzice Ani chcą, żeby ich córka studiować prawo. | syntax | 0 | Ania’s parents want her daughter to study law. |
| Prosimy, żeby rodzice porozmawiać na ten temat z dziećmi. | syntax | 0 | We ask parents to speak with their children about it. |
| Oni są z Francji dlatego mówią tylko po francusku. | preposition | 1 | They are from France, that’s why they speak French. |
| Moja dobra znajoma mieszka w Tokio. | preposition | 1 | My good friend lives in Tokio. |
| Proszę podpisać się na końcu formularza. | preposition | 1 | Please sign at the bottom of the form. |
| Michał i Anka zaczęli się spotykać codziennie po zajęciach. | preposition | 1 | Michał and Anka started meeting everyday after classes. |
| Proces wyrobienia wizy zaczyna się od złożenia wniosku. | preposition | 1 | The process of obtaining visa starts with submitting the request form. |
| Ponad rok temu przeprowadziłem się z Warszawy do Krakowa. | preposition | 1 | I moved from Warsaw to Krakow over a year ago. |
| Nie wiem jak długo pracuje jako sekretarka po tej firmie. | preposition | 0 | I don’t know how long she’s been working as a secretary in this company. |
| Od wykształcenia jestem nauczycielką języka angielskiego. | preposition | 0 | I have English teaching qualifications. |
| W zeszłym roku byliśmy przy wakacjach nad morzem w Grecji. | preposition | 0 | Last year we spent holidays at the seaside in Greece |
| Wszystkie książki stoją za półce ustawione alfabetycznie. | preposition | 0 | All the books on the shelf are in alphabetical order. |
| Nie pamiętam jak ona ma przed imię i nazwisko. | preposition | 0 | I don’t remember her name and surname. |
| Wykłady zaczynają się do jedenastej i kończą o dwunastej. | preposition | 0 | The lectures start at eleven and finish at twelve. |
| Studenci nie będą mogli kupić biletu ze zniżką. | number | 1 | Students won’t be able to buy a discounted ticket. |
| Do sałatki potrzebne są dwa pomidory i jeden ogórek. | number | 1 | For this salad you need two tomatoes and one cucumber. |
| Japończycy nie potrzebują wizy do Polski. | number | 1 | Japanese citizens don’t need a visa to Poland. |
| Każda litera musi być wpisana do oddzielnego pola. | number | 1 | Each letter has to be put in a separate field. |
| Te banany wczoraj były zielone a dziś są żółte. | number | 1 | These bananas were green yesterday and today they are yellow. |
| Lokalne władze potwierdziły doniesienia mediów o ataku hakerów. | number | 1 | The local government has confirmed the media’s information about the hackers’ attack |
| W sklepie Ania stały w kolejce przez pół godziny. | number | 0 | At the shop, Ania spent half an hour in a queue. |
| W grudniu wszystkie dzieci czeka na prezenty od Świętego Mikołaja. | number | 0 | In December all children wait for gifts from Santa. |
| Nasze ostatnie wakacje w Hiszpanii była udane, bo dopisała pogoda. | number | 0 | Our last holiday in Spain went well, because the weather was great. |
| W naszym zespole jest osiem człowieka z różnych krajów. | number | 0 | In our team there are 8 people from different countries. |
| Moja babcia niedawno skończyła sześćdziesiąt roków. | number | 0 | My grandmother has recently had her 60th birthday. |
| Paweł złapał mnie za obie rękę i nie chciał puścić. | number | 0 | Paweł grabbed both my hands and didn’t want to let go. |
| Moi rodzice poznali się na wakacjach w górach. | gender | 1 | My parents met on holiday in the mountains. |
| W Polsce uważa się za niegrzeczne pytanie kobiet ile mają lat. | gender | 1 | In Poland, asking women about their age is considered rude. |
| Sylwia będzie musiała jutro poprosić szefa o urlop. | gender | 1 | Sylwia will have to ask her boss for a leave tomorrow. |
| Zwykle bez mocnej kawy nie ruszam się rano z domu. | gender | 1 | Usually, I don’t leave the house without a strong coffee. |
| Szkoda, że złota polska jesień już się skończyła. | gender | 1 | It’s a shame that the Polish golden autumn has already finished. |
| Według prognozy, w całej Polsce będzie padał jutro śnieg. | gender | 1 | According to the forecast, it will be snowing everywhere in Poland tomorrow. |
| Sylwia i Karolina uczyli się całą noc do egzaminu. | gender | 0 | Sylwia and Karolina have been studied all night for the exam. |
| W wakacje dzieci mieli dużo czasu na zabawę i odpoczynek. | gender | 0 | During holidays, children had a lot of time to rest and play. |
| Cała jego rodzina mieszkali w tym małym mieszkaniu. | gender | 0 | His whole family lived in this small apartament. |
| W nocy będzie niskie temperatura i wysokie ciśnienie. | gender | 0 | At night, the temperature will be low and the pressure high. |
| Londyn to jedno z największa miast na świecie. | gender | 0 | London is one of the biggest cities in the world. |
| Wygląda na to, że warunki do pływania będą w ten weekend wspaniali. | gender | 0 | It seems that the conditions for swimming will be great this weekend. |
| Wiosna zbliża się wielkimi krokami, dlatego warto oglądać prognozę pogody. | conjunction | 1 | The spring is around the corner, that’s why it makes sense to follow the weather forecast. |
| Rzadko chodzę do klubu, bo nie mam z kim tańczyć. | conjunction | 1 | I rarely go clubbing, because I have no one to dance with. |
| Byłam umówiona na wizytę u dentysty, ale okazało się, że nastąpiła pomyłka. | conjunction | 1 | I had an appointment with a dentist but it turned out that there was a misunderstanding. |
| Jej mama pracuje w szkole, a tata w firmie informatycznej. | conjunction | 1 | Her mum works at school and her dad works for an IT company. |
| Nie rozumiem, dlaczego nie chcesz iść z nami do klubu. | conjunction | 1 | I don’t understand why you don’t want to go to the club with us. |
| Nauczę się gotować, jeśli pokażesz mi jak to się robi. | conjunction | 1 | I learn how to cook if you’ll show me how to od it. |
| Uczę się niemieckiego od dwóch lat, jeśli dalej nic nie rozumiem. | conjunction | 0 | I’ve been learning German for two years and I still understand nothing. |
| Nie miałem czasu, albo szybko zjadłem obiad. | conjunction | 0 | I didn’t have time so I ate dinner quickly. |
| Nie znoszę muzyki klasycznej a literatury obcej. | conjunction | 0 | I hate classical music and foreign literature. |
| Poszłam do kina ale obejrzałam naprawdę ciekawy film. | conjunction | 0 | I went to the cinema and watched a really interesting film. |
| Anna chce być szczupła dlaczego codziennie dużo biega. | conjunction | 0 | Anna wants to be slim so she runs a lot everyday. |
| To fajna bluzka bo niestety materiał jest kiepski. | conjunction | 0 | This is a nice blouse, but unfortunately the fabric is bad. |

**SET 2**

| **Sentence** | **Error type** | **Correct** | **Translation** |
| --- | --- | --- | --- |
| Twoje najstarsze dziecko jest bardzo uzdolnione muzycznie. | case | 1 | Your oldest child is musically very talented. |
| Trzeba przyznać, że telefonów są bardzo przydatne. | case | 0 | It must be admitted that phones are very useful. |
| Przywiozę Michałowi parę podkoszulków z nazwą zespołu. | case | 1 | I’ll bring some t-shirts with the band’s name for Michał. |
| Zwykle po pracy brakuje mi motywacją żeby iść na siłownię. | case | 0 | Usually after work I lack the motivation to go to the gym. |
| Wczoraj oddałem mechanikowi samochód do naprawy. | case | 1 | Yesterday I left my car at the garage to be fixed. |
| Myślę, że każdemu Polakiem znana jest ta postać. | case | 0 | I think every Pole knows that person. |
| Problem został rozwiązany przez młodego matematyka. | case | 1 | The problem was solved by a young mathematician. |
| Autobus odjeżdża za dwie minutami z tamtego przystanku. | case | 0 | The bus leaves in two minutes from that bus stop. |
| Dzieci rysowały kredkami po wielkich arkuszach papieru. | case | 1 | The children were drawing with pencils on big sheets of paper. |
| Padał deszcz, więc na wszelki wypadek owinęła książkę gazety. | case | 0 | It was raining, so just in case she wrapped the books in a newspaper. |
| W szufladzie miała schowany długi nóż do listów. | case | 1 | In the drawer she had a long paper knife. |
| Siedzieli w kuchnią i patrzyli na to, co robił kucharz. | case | 0 | They were sitting in the kitchen, watching what the cook was doing. |
| Wczoraj obchodziliśmy osiemnaste urodziny mojej siostry. | set_phrase | 1 | Yesterday we celebrated my sister’s eighteen birthday. |
| Jedziemy dziś na wycieczkę bo świeci słońce i jest piękna pogoda. | set_phrase | 1 | Today we’re going for a trip because the sun is shining and the weather is better |
| Miałeś naprawdę świetny pomysł żeby się dzisiaj spotkać. | set_phrase | 1 | You had a really good idea to meet today. |
| Najlepsze oferty wycieczek można znaleźć w naszym biurze podróży. | set_phrase | 1 | Best trip offers can be found in our travel agency. |
| Jestem pewien, że to nie jego paczka papierosów, bo on nie pali. | set_phrase | 1 | I’m sure it’s not his packet of cigarettes because he doesn’t smoke. |
| Musimy sprawdzić prognozę pogody na poniedziałek i wtorek. | set_phrase | 1 | We have to check the forecast for Monday and Tuesday. |
| Nie zjadłem dziś rano śniadania i usycham z głodu. | set_phrase | 0 | I haven’t eaten breakfast in the morning and now I’m starving. |
| Muszę powstać wcześnie rano żeby zdążyć na pociąg. | set_phrase | 0 | I have to wake up early in the morning to catch the train. |
| W tej nowej sukience leżysz ładnie i elegancko. | set_phrase | 0 | You look pretty and smart in this new dress. |
| Marzy mi się nowy cyfrowy instrument fotograficzny. | set_phrase | 0 | I’m dreaming about a new digital camera. |
| W mieszkaniu nie było kuchni, tylko dodatek kuchenny. | set_phrase | 0 | There was no kitchen in the flat, only a kitchenette. |
| Przyczyna wypadków to nieprzestrzeganie zapisów drogowych. | set_phrase | 0 | Disregarding of the traffic regulations is the reason of the accidents. |
| Widziałem niedawno dom wyceniony na 1000 złotych. | aspect_tense | 1 | I’ve recently seen a housed priced at 1000 złoty. |
| Należy podkreślić, że do obecnego kryzysu przyczyniło się wiele czynników. | aspect_tense | 1 | It must be stressed that many factors contributed to the current crisis. |
| Naszą politykę powinniśmy budować na faktach, a nie na emocjach. | aspect_tense | 1 | Our policy should be build on facts, not emotions. |
| Od nowego roku, duże firmy będą musiały składać specjalne zaświadczenie. | aspect_tense | 1 | From next year, big companies will have to submit a special certificate. |
| Jeśli nie otrzymają takiego certyfikatu narażą się na konsekwencje finansowe. | aspect_tense | 1 | If they won’t receive this certificate they will be financially punished. |
| Z rodziną można spędzać czas na wiele różnych sposobów. | aspect_tense | 1 | There are many ways of spending time with a family. |
| Obrońcy ustawy często przywołali argument trudnych warunków pracy w handlu. | aspect_tense | 0 | The defenders of the bill argued that the working conditions in commerce are hard. |
| Ta część maszyny odpowiedziała za regulację przepływu wody. | aspect_tense | 0 | This part of the machine is responsible for the flow of water. |
| Marysia już godzinę rozwiązała zadanie domowe. | aspect_tense | 0 | Marysia has already spent an hour working on her homework. |
| Wreszcie skończyłem obejrzeć ten serial. | aspect_tense | 0 | I have finally finished watching this series. |
| Lubię od czasu do czasu pić piwo czy dwa. | aspect_tense | 0 | I like to have a beer or two from time to time. |
| Zadzwoniłem do niego wczoraj cały dzień, ale nie odebrał. | aspect_tense | 0 | I tried to call him all day yesterday, but he didn’t pick up. |
| Od początku było widać, że nie byli przygotowani. | syntax | 1 | It was clear from the start that they weren’t prepared. |
| Wstępne wyniki wskazują, że prezydent zdobył 90% głosów. | syntax | 1 | The preliminary results show that the president got 90% of the votes. |
| Joanna zdecydowała, że zapisze się na kurs administracji. | syntax | 1 | Joanna has decided to enroll on an administration course. |
| Pojechałyśmy do galerii handlowej, żeby kupić kilka rzeczy. | syntax | 1 | We went to the shopping centre to buy a few things. |
| Wstałem dziś kwadrans wcześniej, żeby zdążyć na autobus. | syntax | 1 | I woke up a quarter earlier today to catch a bus. |
| Nie mogę znaleźć czasu, żeby umówić się z Pawłem. | syntax | 1 | I can’t find any time to meet with Paweł. |
| Nikt nie mógł uwierzyć, że on przez tyle lat kłamać. | syntax | 0 | Nobody could believe that he was lying for so many years. |
| Mężczyzna zaprzeczył, że to on popełnić morderstwo. | syntax | 0 | The man denied that it was him who committed the murder. |
| Kanapa była tak stara, że już się rozpadać. | syntax | 0 | The couch was so old it started falling apart. |
| Detektyw przyjechał, żeby przesłuchał świadków. | syntax | 0 | The detective came to interrogate the witnesses. |
| Zanim rozpoczęła konferencję poprosiła, żeby nikt im nie przeszkadzać. | syntax | 0 | Before she started the conference, she asked not to be interrupted. |
| Staramy się, żeby nasi widzowie mieć dostęp do najnowszych filmów. | syntax | 0 | We’re trying to provide access to the newest films for our customers. |
| Nie pamiętam o czym rozmawiałyśmy przy śniadaniu. | preposition | 1 | I don’t remember what we talked about during breakfast. |
| Na środku pokoju stał stół a nad nim wisiała lampa. | preposition | 1 | In the middle of the room there was a table and above it there was a lamp. |
| Rano piję kawę z mlekiem, ale bez cukru. | preposition | 1 | In the morning I drink coffee with milk but no sugar. |
| Nie rozumiem w jakim języku oni rozmawiają. | preposition | 1 | I don’t understand the language they speak. |
| Karolina kupiła nową skórzaną torbę za 300 złotych. | preposition | 1 | Karolina bought a new leather bag for 300 złoty. |
| Napisałam do niego maila, ale nic mi nie odpisał. | preposition | 1 | I wrote an email to him, but he didn’t respond. |
| Powinnam kupić nowy album dla moje fotografie. | preposition | 0 | I should buy a new album for my photos. |
| Zajęcia zaczynają się o dziesiątej i kończą przez dwunastej. | preposition | 0 | The classes start at ten and finish at noon. |
| Sklepy z alkoholem są zwykle czynne za całą dobę. | preposition | 0 | The off licence shops are usually open 24/7. |
| Mam straszną ochotę od rurkę z kremem i kawę. | preposition | 0 | I really crave a cream roll and a coffee. |
| W tej restauracji można płacić kartą po bankomatu. | preposition | 0 | You can pay with your ATM card in this restaurant. |
| Na trzydzieste urodziny kupiliśmy mu płytę o polską muzyką klasyczną. | preposition | 0 | For his thirtieth birthday we bought him a CD with Polish classical music. |
| Znajomość języków obcych jest potrzebna w biznesie. | number | 1 | The knowledge of foreign languages is necessary in business. |
| Wszyscy wiedzą, że jest z niego świetny nauczyciel tańca. | number | 1 | Everybody knows he’s a great dance teacher. |
| Biura i urzędy pracują od ósmej do szesnastej. | number | 1 | The offices and bureaus are open from eight to four. |
| Tyle dziś chodziliśmy po mieście, że bolą mnie nogi. | number | 1 | We’ve beed walking around the city for so long that my legs hurt. |
| Zdążyłam wypić dwie mocne kawy przed wyjściem z domu. | number | 1 | I managed to drink two strong coffees before I left the house. |
| Mój tata ma trzy młodsze siostry. | number | 1 | My dad has three younger sisters. |
| Kasia i Tomek pracuje w firmie informatycznej dwa lata. | number | 0 | Kasia and Tomek have been working for an IT company for two years. |
| Wszystkie meble w mieszkaniu byli stare i zakurzone. | number | 0 | All the furniture in the flat were old and dusted. |
| Jesienią i wiosną burze występuje bardzo często. | number | 0 | In the autumn and spring, storms occur very often. |
| Intensywnie uczę się niemieckiego od pięciu rok. | number | 0 | I have been intensely studying German for five years. |
| Dużo starszych osoby nie lubi uczyć się języków obcych. | number | 0 | Many older people don’t like studying foreign languages. |
| Tak długo czytałem ten artykuł, że bolą mnie oko. | number | 0 | I’ve been reading this article for so long my eye hurts. |
| Moi przyjaciele byli pierwszy raz w Polsce i bardzo im się podobało. | gender | 1 | This was the first time my friends were in Poland and they liked it a lot. |
| Mężczyźni przeciętnie żyją dłużej niż kobiety. | gender | 1 | On average, men live longer than women. |
| Michał czekał na nią w restauracji ponad godzinę. | gender | 1 | Michał was waiting for her at the restaurant for over an hour. |
| Wczoraj oglądałam film o argentyńskim tangu. | gender | 1 | Yesterday I watched a film about Argentinian tango. |
| Zarezerwowałem pokój jednoosobowy z łazienką. | gender | 1 | I’ve book a single room with a bathroom. |
| Specjalna komisja wybierze najlepszy projekt. | gender | 1 | A special committee will select the best project. |
| Ania chciał zostać lektorką języka angielskiego. | gender | 0 | Ania wanted to become an English tutor. |
| Do sałatki kucharz wkroiła jeden duży pomidor. | gender | 0 | The cook added one big tomato to the salad. |
| Wczorajsze spotkanie zarządu trwała bardzo długo. | gender | 0 | Yesterday’s board meeting took a long time. |
| Wszyscy wiedzą, że jest z niego świetna nauczyciel tańca. | gender | 0 | Everybody knows he’s a great dance teacher. |
| Pogoda w ten weekend zapowiada się fantastyczny. | gender | 0 | The weather this weekend is supposed to be great. |
| Nie interesują mnie filmy romantyczni ani horrory. | gender | 0 | I’m not interested in romantic comedies or horror films. |
| Ja i moi znajomi wybieramy się do Krakowa, a potem do Warszawy. | conjunction | 1 | My friends and I are going to Krakow and then Warsaw. |
| W weekend możemy albo pojechać w góry albo zostać w domu. | conjunction | 1 | At the weekend we can either go to the mountains or stay home. |
| Dziewczyny poszły na zakupy, więc długo ich nie będzie. | conjunction | 1 | The girls went shopping so they won’t be back for a while. |
| Ty zostajesz po pracy, a my idziemy do klubu. | conjunction | 1 | You’re staying after work and we’re going clubbing. |
| Miałem się z tobą spotkać, ale zapomniałem o wizycie u dentysty. | conjunction | 1 | I was supposed to meet you but I forgot about my dentist appointment. |
| Nie oglądam niemieckiej telewizji, bo nic nie rozumiem. | conjunction | 1 | I don’t watch German TV because I don’t understand anything |
| Proszę posłuchać nagrania, więc następnie uzupełnić tekst. | conjunction | 0 | Please listen to the recording and then fill in the gaps in the text. |
| Nie rozumiem, ale chcesz iść z nami do klubu. | conjunction | 0 | I don’t understand why you don’t want to go to the club with us. |
| Dobrze gram w tenisa, ani lepiej w golfa. | conjunction | 0 | I play tennis well, but I’m better at golf. |
| Do banku jest niedaleko, albo zdążę przed wykładem. | conjunction | 0 | The bank is not far away from here, I’ll get there before the lecture. |
| Data ważności jogurtu minęła wczoraj, jeśli muszę go teraz wyrzucić. | conjunction | 0 | The youghurt expired yesterday so I have to throw it away. |
| Proszę powtórzyć trochę wyraźniej, a niestety nic nie rozumiem. | conjunction | 0 | Please repeat more clearly, unfortunately I don’t understand anything. |
